# Applying NGS Data to Find Evolutionary Network Biomarkers from the Early and Late Stages of Hepatocellular Carcinoma

**DOI:** 10.1155/2015/391475

**Published:** 2015-08-20

**Authors:** Yung-Hao Wong, Chia-Chou Wu, Chih-Lung Lin, Ting-Shou Chen, Tzu-Hao Chang, Bor-Sen Chen

**Affiliations:** ^1^Laboratory of Control and Systems Biology, Department of Electrical Engineering, National Tsing Hua University, Hisnchu 30013, Taiwan; ^2^Biomedical Technology and Device Research Laboratories, Industrial Technology Research Institute, Hsinchu 31040, Taiwan; ^3^Graduate Institute of Biomedical Informatics, College of Medical Science and Technology, Taipei Medical University, Taipei 110, Taiwan

## Abstract

Hepatocellular carcinoma (HCC) is a major liver tumor (~80%), besides hepatoblastomas, angiosarcomas, and cholangiocarcinomas. In this study, we used a systems biology approach to construct protein-protein interaction networks (PPINs) for early-stage and late-stage liver cancer. By comparing the networks of these two stages, we found that the two networks showed some common mechanisms and some significantly different mechanisms. To obtain differential network structures between cancer and noncancer PPINs, we constructed cancer PPIN and noncancer PPIN network structures for the two stages of liver cancer by systems biology method using NGS data from cancer cells and adjacent noncancer cells. Using carcinogenesis relevance values (CRVs), we identified 43 and 80 significant proteins and their PPINs (network markers) for early-stage and late-stage liver cancer. To investigate the evolution of network biomarkers in the carcinogenesis process, a primary pathway analysis showed that common pathways of the early and late stages were those related to ordinary cancer mechanisms. A pathway specific to the early stage was the mismatch repair pathway, while pathways specific to the late stage were the spliceosome pathway, lysine degradation pathway, and progesterone-mediated oocyte maturation pathway. This study provides a new direction for cancer-targeted therapies at different stages.

## 1. Introduction

Cancer is the leading cause of death worldwide, and its etiology occurs at the DNA, RNA, and protein levels. It is a very complex disease involving cascades of spatial and temporal changes in genetic networks and metabolic pathways [1]. Weinberg summarized the important cancer hallmarks [2, 3]. Cancer hallmarks can highlight important cancer mechanisms. In recent years, many systems biology approaches have been applied to study cancers [4–9]. We performed a series of studies on cancer using systems biology approaches. Recently, we have particularly focused on searching for network biomarkers of cancers [10–16].

Hepatocellular carcinoma (HCC) is a major liver tumor (~80%), besides hepatoblastomas, angiosarcomas, and cholangiocarcinomas. Compared to other types of cancer, liver cancer is the third most deadly cancer globally and caused about 700,000 deaths in 2011 [17]. Due to the increasing annual incidences [18] and poor 5-year survival rate (~15%), diagnosis and prognosis of HCC are still important public health issues. Various studies revealed a multistep process involved in liver carcinogenesis [19]; however, the exact biology of HCC remains poorly understood overall [20]. In addition, late confirmation of the occurrence of HCC through traditional histological examinations and tumor sections contributes to the poor survival rate. To accurately diagnose the occurrence of HCC at an early time, it is necessary to understand the cellular and molecular mechanisms of HCC [20]. Hence, in this study, we compared the early and late stages of molecular interaction networks of HCC to reveal the underlying mechanisms of HCC development.

Although the histological and molecular features leading to HCC initiation are still poorly understood, mounting evidence suggests that a gradual accumulation of mutations and genetic changes in hepatocytes, which form the live lobule, may lead to the development of HCC [21]. Exposure to risk factors for liver cancer, such as obesity [22], alcohol addiction, aflatoxin, or hepatitis viruses [21], and the subsequent induction of inflammation may cause advanced hepatocyte necrosis. The recurrence of necrosis and regeneration of hepatocytes provide an opportunity for the introduction of mutations and genetic changes in hepatocytes. During these processes, alternative ways to protect liver lesions occur such as fibrosis and cirrhosis. Although a high correlation between cirrhosis and an HCC diagnosis was reported, the mechanisms of how cirrhosis transforms into HCC are still unknown. Furthermore, after a definite diagnosis of HCC, patients with early-stage liver cancer have better survival rates than those in the late stage. Although HCC's management has substantially changed in the past few decades, the only systemic standard of care for patients with advanced HCC is sorafenib, a multikinase inhibitor, with a mean survival benefit of only 3 months [20, 23]. With advances in affordable, high-throughput technologies, increasing numbers of systems biology studies of HCC diagnoses [24] and treatments [25] have shed light on applications of systems biology to HCC diagnosis, prognosis, and therapy.

As for different etiologies and heterogenic genomic alterations of HCC, the systems biology methodology that integrates Omics data is suitable to develop accurate diagnoses, novel therapeutic targets, and efficient targeted therapies [26]. In this study, NGS data were used to construct protein-protein interaction (PPI) networks (PPINs) of the early and late stages of HCC. The network structure and protein associations of different stages of HCC were compared to obtain a set of significant proteins which play important roles in processes of the progression of HCC.

Chen et al. developed a dynamical network biomarker (DNB) that can serve as a general early-warning signal to indicate an imminent bifurcation or sudden deterioration before the critical transition occurs, which means that it can identify a predisease state using time series microarray data [27–29]. We tried a different approach from their method and used sample microarray data from liver cancer patients at different stages. Our approach could also be extended to predict some similar results as their research. That is, in this study, we simply divided the cancer into early and late stages, but there are more stages of cancer, such as stages I, II, III, and IV. If we could observe the time evolution of cancer biomarkers at these other different stages, we could also predict the predisease state by comparing these cancer biomarkers at different stages.

We reveal the carcinogenesis process from early-stage and late-stage liver cancer. A specific pathway of the early stage was the mismatch repair pathway, while specific pathways of the late stage were the spliceosome pathway, lysine degradation pathway, and progesterone-mediated oocyte maturation pathway.

## 2. Materials and Methods

### 2.1. Overview of the Process for Constructing Liver Cancer Network Markers

**Figure 1:**
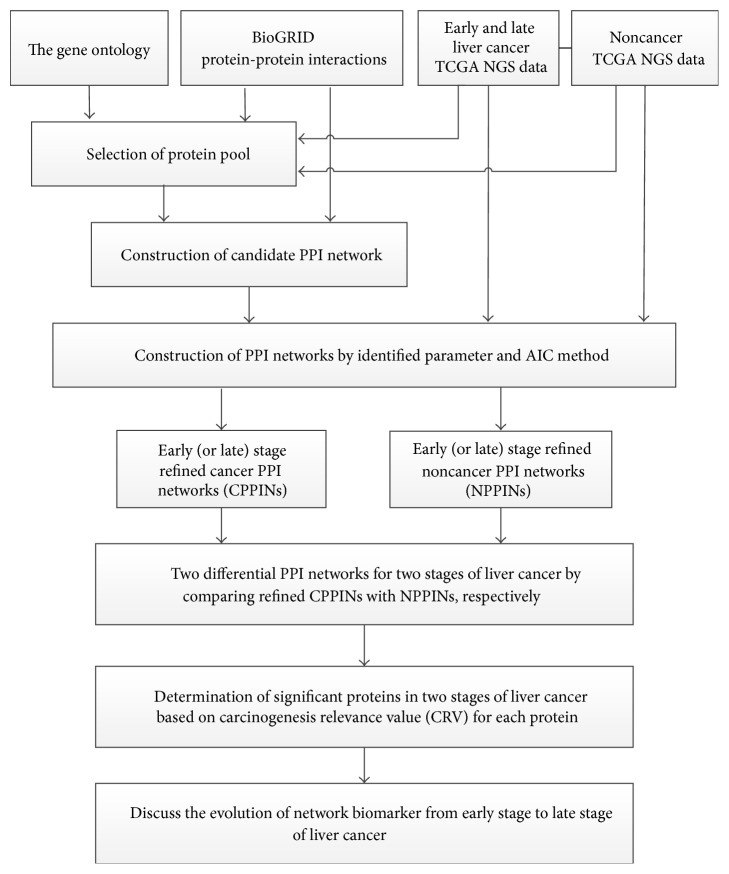
Flowchart illustrating construction of network markers in two stages of liver cancer and the investigation of carcinogenesis mechanisms. We integrated NGS data, the GO database, and protein-protein interaction (PPI) information to construct the PPI network. These data were used for pool selection, and then the selected proteins and NGS data were used to contribute to the PPI network (PPIN) by a maximum-likelihood estimation and model order detection method, resulting in a liver cancer PPIN (CPPIN) and a noncancer PPIN (NPPIN) in the early and late stages of liver cancer. The two constructed PPINs were used to determine significant proteins of tumorigenesis by examining differences between the two PPI matrices of the two constructed PPINs. With the help of a differential PPI matrix (network) between CPPIN and NPPIN, a carcinogenesis relevance value (CRV) was computed for each protein, and significant proteins in carcinogenesis were determined based on *p* values of the CRVs of these proteins in the differential PPI matrix between CPPIN and NPPIN. These significant proteins were obtained for early and late stages of liver cancer.

We successfully used our methods to find core and specific network markers of four different kinds of cancer and the evolution of network markers from the early stage to late stage of liver cancer [15, 16]. A similar theoretical framework was employed in this study to find the evolution of network markers in the early and late stages of liver cancer. The theoretical systems method in this paper was developed from a previous study, but we used a new large dataset of NGS expression data which differ from traditional microarray expression data. A flowchart of the construction of network biomarkers for the early and late stages of liver cancer is shown in Figure 1. We know that a lot of NGS gene expression data have been generated in recent years. Some consider that they are more accurate than traditional microarray expression data. Another key point of this work was to make comparisons with our previous results, such as network biomarkers of liver cancer from microarray data and the evolution of network markers from the early and late stages of liver cancer. We combined two data sources: (1) NGS data of liver cancer and noncancer samples from the GEO database, among which cancer samples were divided into two groups of early-stage and late-stage liver cancer, and (2) the PPI database, which is required to construct PPINs for liver cancer. These data were used for PPI pool selection, and the selected PPIs and microarray data were then used for PPIN construction. Through regression modeling and the maximum-likelihood parameter estimation method, a cancer PPIN (CPPIN) and noncancer PPIN (NPPIN) were obtained. We also constructed a differential PPIN (DPPIN). The constructed CPPIN and NPPIN were compared to obtain a set of significant proteins for liver cancer based on the carcinogenesis relevance value (CRV) for each protein and a statistical assessment. Significant proteins and PPIs among these proteins were used to construct network markers for the early and late stages of liver cancer.

### 2.2. Data Selection and Preprocessing

Liver RNA-seq data were collected from liver HCC (LIHC) of The Cancer Genome Atlas (TCGA) with a batch number of 100. Normalized results, which consisted of reads per kilobase of exon per million mapped reads (RPKM) values, were used to represent gene expressions. The NGS gene expression dataset of liver cancer was obtained from TCGA database. The same dataset contained early-stage and late-stage liver cancer and noncancer samples. We only used data derived from nonprocessed primary biopsies to avoid discrepancies in gene expressions that are intrinsic to cell culture and fixation. Therefore, the dataset utilized contained primary tumor samples of both stages from patients and adjacent nontumor tissue samples from the same cancer patients, which were considered to be control samples. As shown in Table 1, 19 early-stage (stages I and II) and 18 late-stage (stages III and IV) liver HCC tumors and 24 adjacent normal tissue pairs were collected for further analysis. To describe the extent of a patient's cancer, the cancers were classified into four stages according to their degree of invasion and migration using the TNM staging system, as defined by the American Joint Committee on Cancer (AJCC) and the International Union against Cancer (UICC). We then divided the cancer samples into two groups. In general, stages I and II described early-stage cancers that have higher curability rates with medical treatment, while stages III and IV described late stages. We also combined the early and late stages for a total stage. However, there were no corresponding noncancer samples in the surrounding area for each stage, and we had only one group of surrounding noncancer samples (Table 1). The reason why we had to take this step was that NGS samples are harder (and much more expensive) to obtain than microarray data, and the number of samples was small, which may have caused overfitting in the parameter estimation process of our model. So we used the total stage to identify a model to avoid overfitting. We built the CPPIN and NPPIN for early-stage, late-stage, and total-stage liver cancer in this study. We obtained 19 and 18 samples for the early-stage and late-stage liver cancer, respectively, and 24 noncancer samples. Prior to further analysis, the gene expression value, *h*_*ij*_, was normalized to *z*-transformed scores, *g*_*ij*_, for each gene, *i*, and then the resulting normalized expression value had a mean *μ*_*i*_ = 0 and a standard deviation *σ*_*i*_ = 1 for sample *j* [30, 31].

PPI data for* Homo sapiens* were extracted from the Biological General Repository for Interaction Database (BioGRID, downloaded in October 2012). The BioGRID is an open-access archive of genetic and protein interactions that are curated from the primary biomedical literature of all major model organisms. As of September 2012, BioGRID housed more than 500,000 manually annotated interactions from more than 30 model organisms [32]. The above two databases were mined for liver cancer and noncancer PPINs using their corresponding microarray data. These early-stage and late-stage liver cancer and noncancer PPINs were then compared to obtain network markers.

### 2.3. Selection of a Protein Pool and Identification of PPINs for Cancerous and Noncancerous Cells

To integrate gene expressions with PPI data so we could construct the corresponding CPPINs and NPPINs, we set up a protein pool containing differentially expressed proteins. Gene expression values were reasonably assumed to correlate with protein expression levels. We used a one-way analysis of variance (ANOVA) to analyze the expression of each protein and selected proteins with differential expression levels. This method allowed determination of significant differences between cancer and noncancer datasets. The null hypothesis (Ho) was based on the assumption that mean protein expression levels of cancer and noncancer sets were the same. A Bonferroni adjustment [33], a type of multiple testing, was used to detect and correct proteins with a discrepancy. Proteins with a *p* value of <0.01 were included in the protein pool. However, any proteins in the protein pool which did not have PPI information were eliminated. In addition, proteins that were not already in the protein pool were included if their PPI information determined that they were closely associated with proteins already in the pool. As a result, the protein pool contained proteins that had certain differences in expression levels and proteins that had close relationships with the aforementioned proteins.

On the strength of the significant pool and PPI information, candidate PPINs for early-stage and late-stage liver cancer were constructed for liver cancer and noncancer tissues by linking proteins that interacted with each other. In other words, proteins that had PPI information through the pool were linked together, resulting in candidate PPINs.

As the candidate PPIN included all possible PPIs under various environments, different organisms, and experimental conditions, the candidate PPIN needed to be further confirmed by microarray data to identify appropriate PPIs according to the biological processes that are relevant to cancer. To remove false-positive PPIs from each candidate PPIN for different biological conditions, we used both a PPI model identification scheme and a model order detection method to prune each candidate PPIN using the corresponding microarray data to approach the actual PPIN. Here, the PPIs of a target protein *i* in the candidate PPIN can be depicted by the following protein association model:

(1) $$\begin{array}{c} x_{i}\left[\;n\;\right]=\overset{M_{i}}{\underset{j=1}{\sum }}\alpha_{ij}x_{j}\left[\;n\;\right]+\omega_{i}\left[\;n\;\right], \end{array}$$

where *x*_*i*_[*n*] represents the expression level of the target protein *i* for sample *n*; *x*_*j*_[*n*] represents the expression level of the *j*th protein interacting with target protein *i* for sample *n*; *α*_*ij*_ denotes the association interaction ability between the target protein *i* and its *j*th interactive protein; *M*_*i*_ represents the number of proteins interacting with the target protein *i*; and *ω*_*i*_[*n*] represents the stochastic noise due to other factors or model uncertainty. The biological meaning of (1) is that expression levels of target protein *i* are associated with expression levels of proteins that interact with it. Consequently, a protein association (interaction) model for each protein in the protein pool can be built using (1).

After constructing (1) for the PPI model of each protein in the candidate PPIN, we used the maximum-likelihood estimation method [34] to identify association parameters in (1) using microarray data as follows (see Supplementary Materials S.1 available online at http://dx.doi.org/10.1155/2015/391475):

(2) $$\begin{array}{c} x_{i}\left(\;n\;\right)=\overset{M_{i}}{\underset{j=1}{\sum }}{\hat{\alpha }}_{ij}x_{j}\left(\;n\;\right)+w_{i}\left(\;n\;\right), \end{array}$$

where ${\hat{\alpha }}_{ij}$ was identified using microarray data in accordance with the maximum-likelihood estimation method.

Once the association parameters for all proteins in the candidate PPIN were identified for each protein, significant protein associations were determined using the interaction model order detection method based on estimated association abilities, that is, to detect the interaction number *M*_*i*_ in (2). The Akaike information criterion (AIC) [34] and Student's *t*-test [35] were used for both model order selection and significance determination of protein associations in ${\hat{\alpha }}_{ij}$ (see Supplementary Materials S.2).

### 2.4. Determination of Significant Proteins and Their Network Structures in the Carcinogenesis of Liver Cancer

After the interaction number *M*_*i*_′ was determined using the AIC order detection and Student's *t*-test, spurious false-positive PPIs, ${\hat{\alpha }}_{ij}$, in (2) were pruned away, and only significant PPIs that remained were refined as follows:

(3) $$\begin{array}{c} x_{i}\left(\;n\;\right)=\overset{M_{i}^{\prime }}{\underset{j=1}{\sum }}{\hat{\alpha }}_{ij}x_{j}\left(\;n\;\right)+w_{i}^{\prime }\left(\;n\;\right),\;i=1,2,\ldots ,M \end{array}$$

where *M*_*i*_′ ≤ *M*_*i*_ denotes the number of significant PPIs of the PPIN, with the target protein *i*. In other words, a number of *M*_*i*_ − *M*_*i*_′'s (or false positives) were pruned from the PPIs of target protein *i*. One protein by one protein (i.e., *i* = 1,2,…, *M* for all proteins in the refined PPIN in (3)) resulted in the following refined PPIN:

(4) XnAXn+wnXn=x1nx2n⋮xMn,A=α^11…α^1M⋮⋱⋮α^M1⋯α^MM,wn=w1′nw2′n⋮wM′n,

where the interaction matrix *A* denotes the PPIs.

If there was no PPI between proteins *i* and *j* or it was pruned away by the AIC order detection due to insignificance in the refined PPIN, then ${\hat{\alpha }}_{ij}=0$. In general, ${\hat{\alpha }}_{ij}={\hat{\alpha }}_{ji}$, but if this was not the case, the larger one was chosen as ${\hat{\alpha }}_{ij}={\hat{\alpha }}_{ji}$ to avoid the situation where ${\hat{\alpha }}_{ij}\neq {\hat{\alpha }}_{ji}$. The above PPIN construction method was used to construct refined PPINs for each stage of liver cancer (early and late) and noncancer cells. The interaction matrices *A* of the refined PPINs in (4) for cancer and noncancer cells of both the early and late stages of liver cancer were constructed, respectively, as follows:

(5) ACkα^11,Ck…α^1M,Ck⋮⋱⋮α^M1,Ck⋯α^MM,Ck,ANk=α^11,Nk…α^1M,Nk⋮⋱⋮α^M1,Nk⋯α^MM,Nk,

where *k* is the early-stage and late-stage liver cancer; *A*_*C*_^*k*^ and *A*_*N*_^*k*^ denote interaction matrices of the refined PPIN of the *k*th stage of liver cancer and noncancer, respectively; and *M* is the number of proteins in the refined PPIN. Therefore, the protein association model for CPPIN and NPPIN in the *k*th-stage liver cancer and noncancer samples can be represented by the following equations according to (4) and (5):

(6) xCknACkxCn+wCknxNkn=ANkxNn+wNkn,

where *k* is early-stage and late-stage liver cancer, *x*_*C*_^*k*^(*n*) = [*x*_1*C*_^*k*^ *x*_2*C*_^*k*^ ⋯ *x*_*MC*_^*k*^]^*T*^ and *x*_*N*_^*k*^(*n*) = [*x*_1*N*_^*k*^ *x*_2*N*_^*k*^ ⋯ *x*_*MN*_^*k*^]^*T*^ denote vectors of expression levels, and *w*_*C*_^*K*^(*n*) and *w*_*N*_^*K*^(*n*) indicate noise vectors of the PPINs in the *k*th stage liver cancer and noncancer cells, respectively.

The different matrix *A*_*C*_^*k*^ − *A*_*N*_^*k*^ of the differential PPI network between CPPIN and NPPIN in the *k*th stage liver cancer is defined as follows:

(7) Dkd11k…d1Mk⋮⋱⋮dM1k⋯dMMk=α^k11,C−α^k11,N…α^k1M,C−α^k1M,N⋮⋱⋮α^kM1,C−α^kM1,N⋯α^MM,Ck−α^MM,Nk,

where *k* is early-stage and late-stage liver cancer, *d*_*ij*_^*k*^ denotes the protein association ability difference between CPPIN and NPPIN in the *k*th-stage liver cancer, and matrix *D* ^*k*^ indicates the difference in network structures between CPPIN and NPPIN in the *k*th-stage liver cancer. In order to investigate carcinogenesis from the difference matrix, *D* ^*k*^, between CPPIN and NPPIN of the *k*th-stage liver cancer in (7), a score, which we called the carcinogenesis relevance value (CRV), was presented to quantify the correlation of each protein in *D* ^*k*^ with the significance of carcinogenesis as follows [30]:

(8) $$\begin{array}{c} CRV^{k}=\left[\begin{array}{c} CRV_{1}^{k}\\ \vdots \\ CRV_{i}^{k}\\ \vdots \\ CRV_{M}^{k} \end{array}\right], \end{array}$$

where CRV_*i*_^*k*^ = ∑_*j*=1_^*M*^|*d*_*ij*_^*k*^| and *k* is early-stage and late-stage liver cancer.

The CRV_*i*_^*k*^ in (8) quantifies the differential extent of protein associations of the *i*th protein (the absolute sum of the *i*th row of *D* ^*k*^ in (7)) and the CRV^*k*^ can differentiate CPPIN from NPPIN in *k*th-stage liver cancer. In other words, the CRV_*i*_^*k*^ in (8) represents the network structure difference of the *i*th protein between cancer and noncancer networks in the *k*th-stage liver cancer.

In order to investigate what proteins are more likely involved in *k*th-stage liver cancer, we needed to calculate the corresponding empirical *p* value to determine the statistical significance of the CRV_*i*_^*k*^. To determine the observed *p* value of each CRV_*i*_^*k*^, we repeatedly permuted the network structure of the candidate PPIN of the *k*th-stage liver cancer as a random network of *k*th-stage liver cancer. Each protein in the random network of the *k*th-stage liver cancer had its own CRV to generate a distribution of CRV_*i*_^*k*^ for *k* = early-stage and late-stage liver cancer. Although the network structure was randomly disarranged, linkages of each protein were maintained. In other words, proteins with which a particular protein interacted were permuted without changing the total number of protein interactions. This procedure was repeated 10^5^ times, and the corresponding *p* value was calculated as the fraction of the random network structure in which the CRV_*i*_^*k*^ was at least as large as the CRV of the real network structure. According to distributions of the CRV_*i*_^*k*^ of the random networks, the CRV_*i*_^*k*^ in (8) with a *p* value of ≤0.01 was regarded as a significant CRV, and the corresponding protein was determined to be a significant protein in the carcinogenesis of the *k*th-stage liver cancer: a protein with a *p* value of >0.01 was removed from the list of significant proteins in carcinogenesis (in other words, if the *p* value of CRV_*i*_^*k*^ was >0.01, then the *i*th protein was removed from the CRV_*i*_^*k*^ in (8), and the remainder in the CRV^*k*^ with *p* values of CRVs of <0.01 was considered significant proteins of the *k*th-stage liver cancer).

Based on *p* values of the CRVs for all proteins (*i* = 1,2,…, *M*) and the two stages of liver cancer (*k* = early-stage and late-stage liver cancer), we generated two lists of significant proteins for each of the two stages according to the CRV and a statistical assessment of each significant protein in the CRV^*k*^ in (8). We found 152 significant proteins in the early-stage liver cancer and 50 significant proteins in the late-stage liver cancer. These proteins showed significant changes between the CPPIN and NPPIN in the carcinogenic process according to their corresponding stage of cancer, and we suspected that these changes might play important roles in the carcinogenesis process of liver cancer. These findings warrant further investigation.

Intersections of these significant proteins in the early and late stages of liver cancer and their PPIs are known as the core network markers appearing in all stages of liver cancer. In contrast, unique significant proteins and their PPIs in each stage of liver cancer are known as specific network markers for each stage of cancer. We found 18 significant proteins that could be classified as core network markers over the entire carcinogenesis process of liver cancer. We also found 134 significant proteins as specific network markers of early-stage liver cancer and 32 significant proteins as specific network markers of late-stage liver cancer.

### 2.5. Pathway Analysis

Much valuable cellular information can be found using known pathways, which are useful for describing most “normal” biological phenomena. All of these known pathways are the result of repeated testing and verification, and the entire pathway network has defined most links. Therefore, the proteins we identified to be significant in the above network markers were mapped onto known pathway networks (e.g., the Kyoto Encyclopedia of Genes and Genomes (KEGG) and PANTHER pathways) to investigate significant pathways with network markers and explore relationships between these pathways and the carcinogenesis of liver cancer. This approach supports the view that systems biology can help identify significant network biomarkers in both normal and cancerous pathways and their roles in the pathogenesis of cancer.

Together with comprehensive pathway databases such as the KEGG, we used a series of bioinformatics pathway analytical tools to identify biologically relevant pathway networks [36]. The KEGG includes manually curated biological pathways that cover three main categories: systems information (e.g., human diseases and drugs), genomics information (e.g., gene catalogs and sequence similarities), and chemical information (e.g., metabolites and biochemical reactions). At present, the KEGG contains 134,511 distinct pathways generated from 391 original reference pathways [37]. Therefore, to investigate pathways involved in carcinogenesis, the bioinformatics database, DAVID [38, 39], which generates automatic outputs of the results from a KEGG pathway analysis [38], was used for the pathway analysis of significant proteins identified as network markers to determine their roles in the pathogenesis of early- and late-stage liver cancer. Our methodology did not include a pathway analysis or gene set enrichment analysis (GSEA). To complete our research results, we used NOA software for the pathway analysis and GSEA of biological processes, cellular components, and molecular functions [40, 41].

### 2.6. Explore the Evolution of Network Biomarkers in the Liver Carcinogenesis via PPI Network through NGS Data

Our cancer PPI model was constructed from the differential expression of cancer and noncancer microarray data and data mining of PPI information from the BioGRID database. So, the early-stage and late-stage liver CPPINs and NPPINs were the results of our systems biology model using the original NGS data and PPI databases. There were two key factors that affected our final results.

*(i) The Effect of Different Original PPI Databases*. We know that PPI databases, such as the BioGRID and MIPS, are constructed from putative samples and validated by wet-lab experiments. Due to advances in many high-throughput experimental skills, the original PPI databases have evolved over time. The newly updated original PPI databases are the second factor to affect the final results.

**Figure 2:**
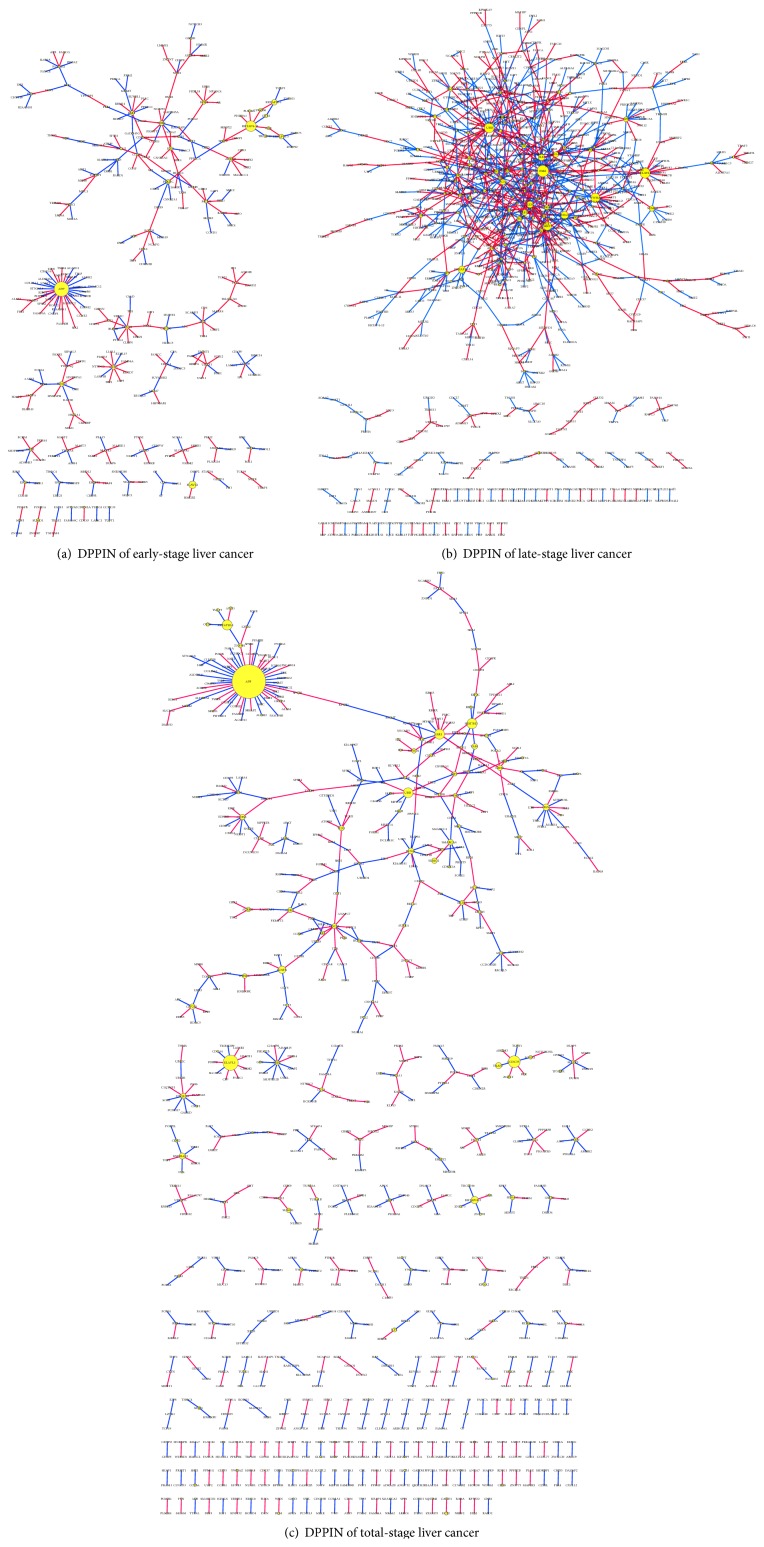
The constructed cancer differential protein-protein interaction networks (DPPINs) for early, late, and total stages of liver cancer. This figure shows the DPPINs with edge and node information for the early, late, and total stages of liver cancer. The DPPIN is the difference between the cancer PPIN (CPPIN) and noncancer PPIN (NPPIN). The figures were created using Cytoscape.

*(ii) The Effect of the Systems Biology Model*. Microarray data, PPI databases, and the PPI interaction model in (1) were employed to construct PPI networks of normal and cancer cells by the maximum-likelihood parameter estimation method (see Supplementary Materials S.1). The AIC system order detection method (Supplementary Materials S.2) was used to prune false-positive PPIs to obtain actual PPI networks of normal and cancer cells; that is, we used the so-called reverse engineering method to construct PPI networks of normal and cancer cells. Then, the DPPIN between the CPPIN and NPPIN was obtained in (7) to investigate PPI variations of each protein in the DPPIN due to carcinogenesis. Finally, the CRV based on PPI variations was also proposed to evaluate the significance of carcinogenesis for each protein of the DPPIN. Proteins with a significant CRV (*p* < 0.01) were considered significant proteins of the cancer. Significant proteins in Table 3 were these significant proteins of early-stage and late-stage liver cancer, and these proteins and their PPIs were used to construct the PPI network in Figure 2. Finally, from early-stage and late-stage liver cancer network markers, we investigated mechanisms of the carcinogenesis process with the help of databases (e.g., the GO database and the DAVID and KEGG pathway databases) to find multiple network targets for cancer therapy. Unlike conventional theoretical methods which always give a single mathematical model for a cancer network for a more-detailed theoretical analysis, this study introduced a systems biology approach to cancer network markers based on actual NGS data through so-called reverse engineering, a systems statistical method, and a data mining method in combination with large databases. These are the novelty and significance of our study. Although we described the novelty of our systems biology model, we have validated our results by a literature survey of research. In the future, our results can be validated by other researchers' wet-lab experiments, and we will repeatedly modify our mathematical system model. This is the third key factor that affected our results. Although not directly, it also influenced the protein interaction network.

We also know that biosystems evolve with time. It is obvious that early-stage and late-stage patients have very different symptoms; these are key features we used to classify early-stage and late-stage liver cancer. Since liver cancer patients in the two stages have very different symptoms, the NGS data of these two stages of patients should undoubtedly greatly differ. As described above, protein expressions from NGS data are one of the key factors of our systems biology model producing the final CPPINs and NPPINs, and the CPPINs and NPPINs gave the final network biomarkers from our systems biology model. So, the most important thing for evolution of network biomarkers is evolution of the NGS data in both stages of liver cancer, which is inherent in the exhibition of cancer-related genes due to DNA mutations in the carcinogenesis process.

## 3. Results and Discussion

### 3.1. Evolution of Network Biomarkers in Early-Stage and Late-Stage Liver Cancer

We built the DPPIN to examine the early, late, and total stages of liver cancer (Figure 2). CRVs of each protein in the three networks were calculated. This figure shows more-detailed information than just the CRVs; that is, it shows the changes of edges and nodes at different stages of the network. By screening using *p* values of CRVs, we found significant proteins of network markers for these three stages of liver cancer. Similar to our previous experience with bladder cancer [16], we wanted to reveal the carcinogenesis mechanisms of liver cancer in the early and late stages. Because we combined the early stage (stages I and II) and late stage (stages III and IV) into a total stage (stages I~IV), it is not surprising that most of the pathways associated with either the early or late stage overlapped those of the total stage. We had to perform this step because NGS samples are more difficult (and much more expensive) to obtain than microarray data; that is, there were few samples. This shortage may have caused overfitting in the parameter estimation process of our model in (2). So we used the total stage again to build a model to avoid overfitting. To summarize, we built a model using early, late, and total stages. Numbers of samples in these three stages were 19, 18, and 37, respectively (37 being the sum of 19 and 18). The reason for using the total stage is that 19 and 18 are small numbers of samples, and there was the chance that overfitting may have occurred. So we used the sum of the early and late stages (37) to see the entire picture between cancer and noncancer tissues.

### 3.2. Network Markers of Early, Late, and Total (Early Plus Late Stages) Stages of Liver Cancer

After *p* value (0.01) screening, we found that there were 43, 80, and 74 significant proteins for the early, late, and total stages of liver cancer, respectively. In addition, their corresponding CRVs ranged 6.0~46.5, 6.1~34.1 and 6.5~81.5, respectively. These significant proteins and their PPIs were used to construct network markers for the early, late, and total stages of liver cancer. The intersection network of markers of the early and late stages was a core feature that contained 27 significant proteins associated with carcinogenesis. We list the 27 significant proteins and their corresponding CRVs and *p* values in both stages of liver cancer in Table 2. In the second step, we separately list the top 20 significant proteins in the early and late stages of liver cancer in Table 3. The full list of the 43, 80, and 74 significant proteins for these three stages of liver cancer is given in Supplementary Tables S3.  Table 4 shows the top 30 proteins of the total stage.  Table 5 shows interactions with our previous results using microarray data.

### 3.3. Pathway Analysis of the Total Stage of Liver Cancer

**Figure 3:**
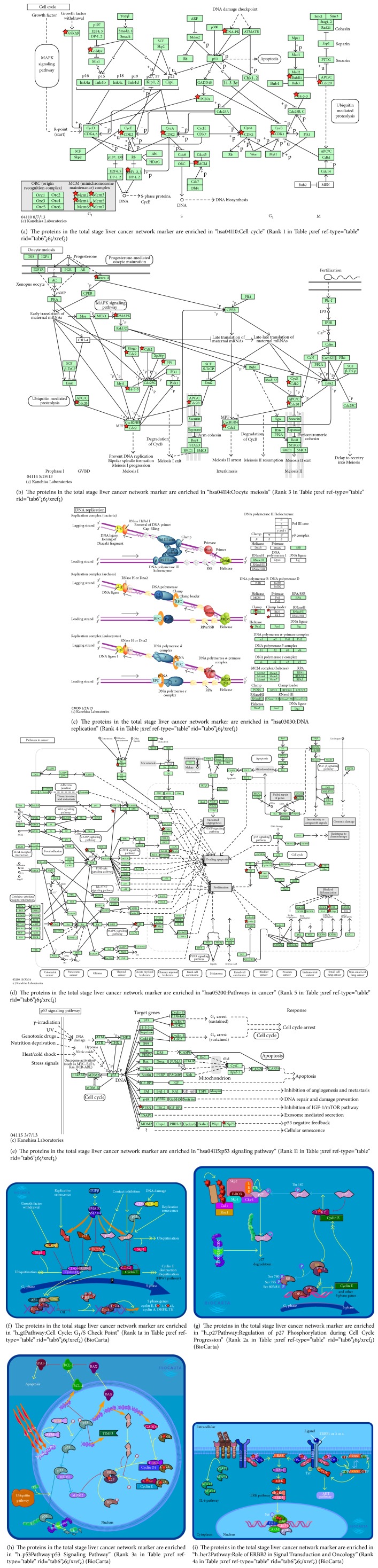
Overview of significant pathways in network marker of total stage of liver cancer. Among KEGG pathways identified via the DAVID tool (Table 4) showing significant associations with specific proteins of the early stage of liver cancer, these molecular pathways had *p* values of ≤0.05. This indicates that these pathways play important roles in the carcinogenesis mechanism in the early stage of liver cancer. The network markers of the early stage of liver cancer highlighted by stars show potentially targets in the pathways. Due to the different naming systems, the same proteins in these tables and in the text are labeled with different names.

**Figure 4:**
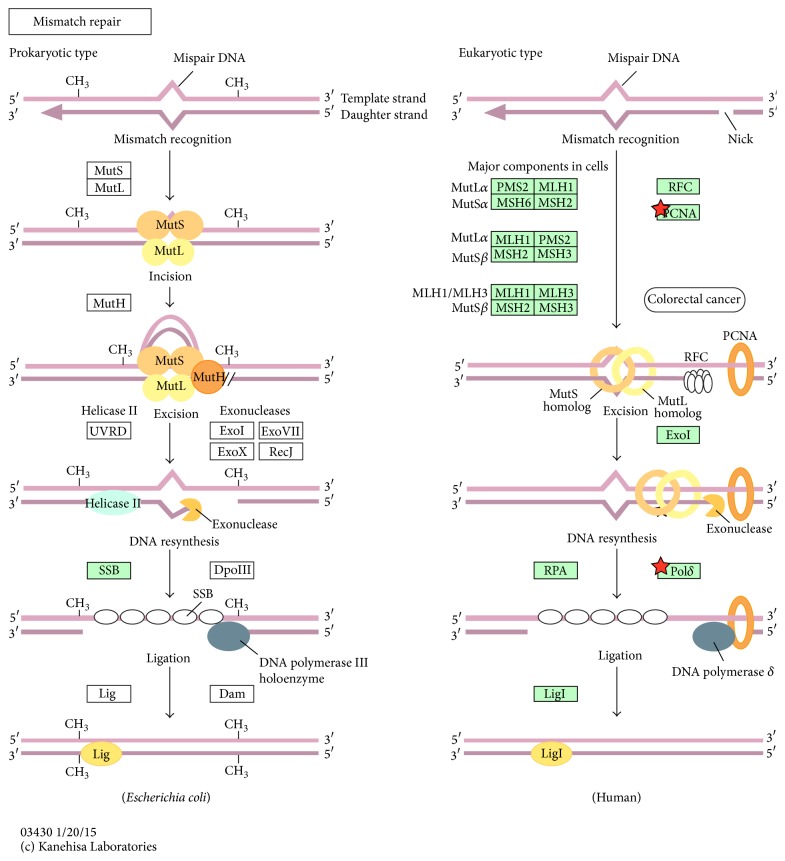
Overview of significant pathways of network markers of early-stage liver cancer. KEGG pathways accessed using the DAVID tool (Table 7) show significant associations with specific proteins in the early stage of liver cancer; these molecular pathways are labeled with *p* values of ≤0.05. These pathways were shown to play important roles in the carcinogenesis mechanism in the early stage of liver cancer. Network markers of the early stage of liver cancer as highlighted by stars show potential targets in the pathways. Due to different naming systems, the same proteins in the tables and text have different names. The proteins in the early-stage liver cancer network marker are enriched in “hsa03040:Mismatch repair” (Rank 9 in Table 7).

**Figure 5:**
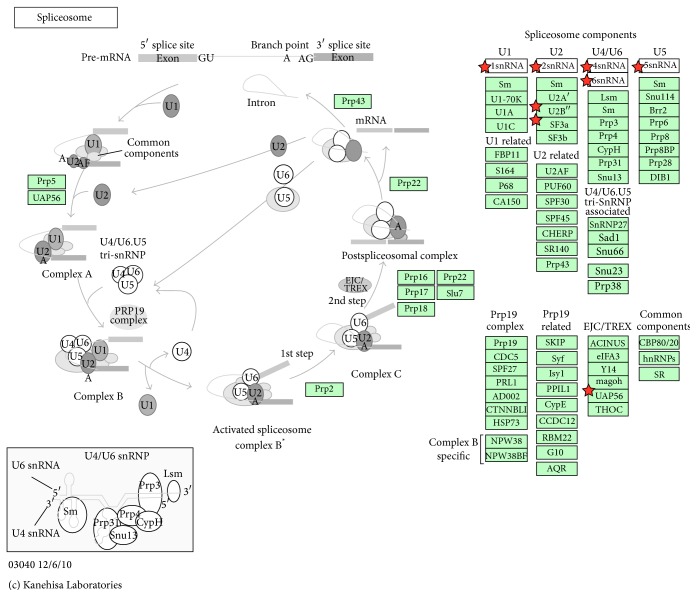
Overview of significant pathways of network markers of late-stage liver cancer. KEGG pathways identified using the DAVID tool (Table 8) show significant associations with specific proteins in the late stage of liver cancer; these molecular pathways are labeled with *p* values of ≤0.05. These pathways were shown to play important roles in the carcinogenesis mechanism of the early stage of liver cancer. Proteins in network markers of the early stage of liver cancer as highlighted by stars are potential targets in the pathways. Due to different naming systems, the same proteins in the tables and in the text have different names. The proteins in the total stage liver cancer network marker are enriched in “hsa03040:Spliceosome” (Rank 5 in Table 8).

We first analyzed the pathway of the total stage of liver cancer using the David database. As stated above, the key point of this research was to find evolutionary mechanisms of liver cancer from the early and late stages, but the number of samples of NGS data was small, so we had to combine the early and late stages to see the overall picture of the liver cancer network. The five key pathways we were interested in, which were selected by these 74 key proteins, are listed as follows: (1) 14 proteins in hsa04110 were associated with the cell cycle (Figure 3(a)), (2) seven proteins in hsa04114 were associated with oocyte meiosis (Figure 3(b)), (3) five proteins in hsa03030 were associated with DNA replication (Figure 3(c)), (4) 10 proteins in hsa05200 were associated with cancer pathways (Figure 3(d)), and (5) four proteins in hsa04115 were associated with the p53 signaling pathway (Figure 3(e)). We only focused on those pathways related to liver cancer and eliminated unrelated ones, such as prostate cancer, glioma (brain cancer) pathway, and small cell lung cancer pathway (Table 6).

*(1) Cell Cycle*. An abnormal cell cycle is highly related to cancer mechanisms. We described this in our previous study [15, 16]. More clues and mechanisms were revealed in this study, so we have to repeat and highlight the previous results while adding the new results. Dysregulation of the cell cycle governs deviant cell proliferation in cancer. Loss of the ability to control cell cycle checkpoints induces abnormal genetic instability. It is well known that the cell cycle is composed of two consecutive periods, characterized by DNA replication, and of sequential differential and segregation of replicated chromosomes into two separate daughter cells. This may be due to activation of tumorigenic mutations, which were recognized in various tumors at different levels in mitogenic signal transduction pathways: (1) ligands and receptors (receptor mutations of HER2/neu [ErB2] or amplification of the HER2 gene), (2) downstream signal transduction networks (Raf/Ras/MAPK or PI3K-AKT-mTOR), and (3) regulatory genes of the cell cycle (cyclin D1/CDK4, CDK6, and cyclin E/CDK2) [42]. There are many reported discussions regarding cell cycle regulators and checkpoint functions involved in HCC [43–46]. Herein, 14 of the total stage network markers identified were involved in the cell cycle pathway. In this case, we found that five of these 14 markers were related to the DNA replication pathway. This shows that the cell cycle and DNA replication are highly related to liver cancer at the total stage.

Because the cell cycle is so crucial to cancer, we list other pathways given by BioCarta (Figures 3(e) and 3(f)). The former shows that these key proteins are related to the G_1_/S checkpoint, while the later shows that they are related to regulation of p27 phosphorylation during cell cycle progression.

Dituri et al. showed that changes in the cell cycle checkpoint frequently occur during HCC. They identified different pathways from us and showed that the phosphatidylinositol-3-kinase (PI3K)/protein kinase B (AKT)/mammalian target of the rapamycin (mTOR) pathway is the key pathway for HCC. They used the human PLC/PRF/5, Hep3B, HepG2, HLE, and HLF HCC cell lines and a normal human hepatocyte cell line. Although they and our group did not identify the same targets, the key point is that an abnormal cell cycle is a complex mechanism. Their scope covered the G_0_-G_1_, G_2_-M, and G_1_-S phases [45]. Our future work will try to link our network makers with mechanisms discovered by other groups.

Furuta et al. showed that micro- (mi)RNAs are key posttranscriptional regulators of gene expression and are usually deregulated in HCC. They identified four miRNAs, mir-101, mir-195, mir-378, and mir-497 that are always silenced in HCC. In this research, we did not include miRNAs when building the model [46]. We know that miRNAs play significant roles in genetic regulatory networks, and our future work will incorporate miRNAs into our model to reveal more hidden mechanisms of HCC.

*(2) Oocyte Meiosis*. We did notidentify this pathway in our previous study. Zhang et al. used network-based bioinformatics methods to identify biomarkers for HCC and identified this pathway with 19 upregulated genes with a *p* value of 0.0016 [47]. We identified seven genes in this pathway with a very low *p* value of 8.70*E*−05. Terret et al. claimed that mouse oocytes are a paradigm of cancer cells. They showed asymmetric division in both mitotic cells and oocytes cells, and this causes cancer cells to divide [48].

*(3) DNA Replication*. As we stated above, we identified five genes of the DNA replication pathway which are also involved in the cell cycle pathway. Macheret and Halazonetis claimed that DNA replication stress is a hallmark of cancer [49], so we have to discuss this topic in more detail here. Hanahan and Weinberg published two review papers on the topic of cancer hallmarks, and the key points are listed here. Based on their first-generation cancer hallmarks [2], Hanahan and Weinberg extended their work to second-generation hallmarks of cancers [3]. It inspired many people and stimulated many ideas on cancer targeted therapies.

*(i) Sustaining Proliferative Signaling*. Cancer cells can sustain proliferative signaling in various ways, become independent of exogenous growth signals, and so can proliferate. They also have the ability to generate growth factor ligands themselves.

*(ii) Evading Growth Suppressors*. Cancer cells can ignore signals that inhibit cell proliferation. Retinoblastoma-associated (RB) and TP53 proteins are the two main prototypes of tumor suppressors. They are also important targets of cancer therapies.

*(iii) Activating Invasion and Metastasis*. Invasion and metastasis comprise multistep processes, usually called the invasion-metastasis cascade. This cascade is related to the mechanisms of miRNAs [50, 51].

*(iv) Enabling Replicative Immortality*. Cancer cells becoming immortal is a lethal event for cancer patients. There are three important topics to this immortality: reassessing replicative senescence, delayed activation of telomerase, and new noncanonical functions of telomerases.

*(v) Inducing Angiogenesis*. Both invasion and metastasis need new-born blood vessels to supply nutrients for cancer cells.

*(vi) Resisting Cell Death*. Cancer cells have many strategies to disable apoptosis. Cancer cells becoming immortal is another lethal event for cancer patients.

When we tried to apply a systems biology approach to cancer therapy, understanding cancer hallmarks was the most important and basic step. There are many investigations of cancer systems biology based on Weinberg's work. Negrini et al. discussed the genomic instability characteristics of cancers and evolving hallmarks of cancer [52]. Hornberg et al. used the concept of complex signaling networks and increases in complexity to directly name cancers as systems biology disease [53]. Barillot et al. merged the six hallmarks into the following three main properties related to the proliferation, survival, and dissemination of cancers. They are the defective control of the cell cycle, defective control of cell death, and the invasiveness and metastatic potential. There are three key levels at which normal cells transform into cancer cells: mutations of DNA, transcription of DNA to RNA, and RNA translation to proteins. Hallmarks are omens and portent of cancers, and, by a pathway analysis, we can find drug targets at this stage.

Cancer is a network disease, that is, a dysregulation of the entire network. Determining how to build a cancer network is the first important task.

We have briefly reviewed and summarized the key points of Weinberg's theory. Now we focus on DNA replication stress, because it was identified as another hallmark of cancer. Macheret and Halazonetis claimed that the sustained proliferation hallmark can be regarded as mutations in oncogenes and tumor suppressor genes that are involved in the cell growth pathway. Mutations of TP53, ATM, or MDM2 genes can allow escaping from the apoptosis hallmark. He discussed oncogene-induced DNA replication stress and the role it plays as a cancer progression driver [49].

*(4) p53 Signaling Pathway*. Soussi et al. discussed a multifactorial analysis of p53 alterations in human cancers [54]. Ueda et al. claimed that the functional inactivation but not the structural mutation of p53 can cause liver cancer [55].

### 3.4. Pathway Analysis of Early-Stage Liver Cancer

Four of these five key pathways discussed in the total stage (cell cycle, DNA replication, oocyte meiosis, and p53 signaling) were also selected by both early and late stages, although the proteins involved in these pathways were not the same (Table 7). We should pay more attention to the evolution from the early to late stages.

*(1) Mismatch Repair*. This is a key pathway that was only selected by the early stage (with higher *p* value of 0.09) but not the late stage (Figure 4). Fujimoto et al. sequenced the entire genome of liver cancers to identify etiological influences on mutation patterns. They discussed transcription-coupled repair and mismatch repair-deficient tumors to show that mismatch repair plays a significant role in liver cancer [56].

### 3.5. Pathway Analysis of Late-Stage Liver Cancer

*(1) Spliceosome*. It is important that we selected the spliceosome pathway again in late-stage liver cancer (Table 8), because we selected it in our previous results of late-stage bladder cancer. We review previous results of the spliceosome pathway [16] and then add new results here. Alternative splicing is a modification of premessenger (m)RNA transcripts in which internal noncoding regions of pre-mRNA (introns) are removed and then the remaining segments (exons) are joined (Figure 5). The formation of mature mRNA is subsequently capped at its 5′ end, polyadenylated at its 3′ end, and transported out of the nucleus to be translated into proteins in the cytoplasm. Most genes use alternative splicing to generate multiple spliced transcripts. These transcripts contain various combinations of exons resulting from different mRNA variants, and these are synthesized as protein isoforms. Exons are always around 50~250 base pairs, whereas introns can be as long as several thousands of base pairs. For nuclear-encoded genes, splicing takes place within the nucleus simultaneously with or after transcription. Splicing is necessary for eukaryotic mRNA before it can be translated into the correct protein. The spliceosome is a dynamic intracellular macromolecular complex of multiple proteins and ribonucleoproteins (RNPs). For many eukaryotic introns, the spliceosome carries out the two main functions of alternative splicing: first, it recognizes intron-exon boundaries, and, second, it catalyzes cut-and-paste reactions that remove introns and concatenate exons. Various spliceosomal machinery complexes are formed from five RNP subunits, termed uridine- (U) rich small nuclear (sn)RNPs, that are transiently associated with more than 760 non-snRNP splicing factors (RNA helicases, SR splicing factors, etc.) [57, 58]. Each spliceosomal snRNP (U1, U2, U4, U5, and U6) consists of a U-rich snRNA complexed with a set of seven proteins known as canonical Sm core or SNRP proteins. The seven Sm proteins (B/B′, D1, D2, D3, E, F, and G) form a core ring structure that surrounds the RNA. All Sm proteins contain a conserved sequence motif in two segments (Sm1 and Sm2) that are responsible for the assembly and ordering of snRNAs. They form the Sm core of spliceosomal snRNPs [59] and process pre-mRNA [60]. Spliceosomes not only catalyze splicing by a series of reactions, but also are the main cellular machinery that guides splicing. Recently, scientists found two natural compounds that can interfere with spliceosome function that also display anticancer activity in vitro and in vivo [61, 62]. Therefore, it is thought that inhibiting the spliceosome can be a new target for anticancer drug development [63], and it should be validated in vivo and in vitro in the future [56].

Tian et al. used a phosphoproteomic analytical method on the highly metastatic HCC cell line, MHCC97-H. They reported that phosphoproteins were also found in the spliceosome pathway, and they were related to liver cancer [64].

*(2) Progesterone-Mediated Oocyte Maturation*. Zhang et al. also identified this pathway [47].

*(3) Lysine Degradation*. Huang et al. identified this pathway in liver cancer [65].

### 3.6. Comparison with Our Previous Liver Cancer Network Biomarkers Using a Microarray Analysis

We found 15 common proteins in this study and our previous results (Table 5). That tells us that the NGS is a revolutionary technology, but it can be trusted to not give totally different results from microarray data.

### 3.7. Comparison of Microarray and NGS Technology

Gene expression profiling by microarray has been successful at demonstrating the patterns of mRNAs within tissues and cells. Although microarray platforms showed similar levels of concordance with the RNA-seq data, the next generation sequencing (NGS) technologies provided high sensitivity, specificity, and accuracy as compared to the microarray platforms [66]. Due to NGS providing a more detailed observation at the transcriptome, scientists and biologists have been eager to apply NGS for gene expression profiling. Microarrays only return results from those regions for probes which have been designed. Therefore, microarrays are only as good as the databases from which they are designed. NGS methods provide aspects of the transcriptome without a priori knowledge, allowing for the analysis and discovery of novel transcripts, noncoding RNAs, and alternative splicing. Thus, RNA sequencing methods enable the most accurate detection and quantification for transcriptome analysis.

## 4. Conclusions

Liver cancer is the third most deadly cancer causing about 700,000 deaths in 2011 worldwide. It is a lethal disease like other cancers. There are three important topics in this research. The first was to compare this study with our previous research of liver cancer microarray data, because the microarray technology may someday be replaced by the NGS technology. We found the results to be good, because there were a lot of key proteins identified by both methods. The second was to reveal the carcinogenesis process from the early to late stages of liver cancer. The specific pathway of the early stage was the mismatch repair pathway, and the specific pathways of the late stage were the spliceosome pathway, lysine degradation pathway, and progesterone-mediated oocyte maturation pathway. This suggests novel directions for choosing different targeted therapeutic strategies at different stages of cancer. In particular, compared to our previous results of bladder cancer, we found that the spliceosome pathway is a significant pathway in the late stage of both liver cancer and bladder cancer. Our future work will focus greater attention on this pathway related to various cancers and consider it as a new drug target for anticancer therapies.

## Supplementary Material

Supplementary material S.1 uses the Maximum Likelihood Method to do the parameter identification of regression model in equation (1). S.2 uses the AIC and student's t-test to calculate the p-values of association abilities, and detect the system model order and determine the significance of the model parameters. Table S3: (a) The 43 identified significant proteins of early stage liver cancer. (b) The 80 identified significant proteins of late stage liver cancer. (c) The 74 identified significant proteins of total stage liver cancer.

## Figures and Tables

**Table 1:** Descriptive information on datasets extracted from the TCGA database used in this study. Cases are grouped by the type of cancer, and surrounding normal tissues came from human patients in the early or late stage of liver cancer.

Cancer type	TCGA dataset number	Early stage sample#	Late stage sample#	Normal sample#	Platform
Liver cancer	Batch100 of LIHC	19	18	24	Illumina HiSeq 2000

**Table 2:** The 27 identified significant proteins of core network marker in both early-stage and late-stage liver cancer (intersection).

Common network marker of early-stage and late-stage liver cancer					
Protein	CRV	*p* value	Cancer_AvgExp	Control_AvgExp	log_2_⁡FC
APP	46.52	0.00001	13724	14041	−0.03
APP	13.87	0.00066	15083	14041	0.1
ELAVL1	22.99	0.00033	1725	1319	0.39
ELAVL1	29.69	0.00003	1791	1319	0.44
KRTAP10-5	14.73	0.00098	1	1	0.04
KRTAP10-5	20.54	0.00022	1	1	0.06
H2AFX	14.08	0.00105	608	271	1.17
H2AFX	15.43	0.00053	806	271	1.58
CDK1	13.69	0.00108	335	34	3.32
CDK1	10.31	0.00119	596	34	4.15
ESR1	12.81	0.00119	228	1537	−2.76
ESR1	34.1	0.00000	245	1537	−2.65
EZH2	12.22	0.00127	310	49	2.66
EZH2	19.05	0.00027	394	49	3
CEP250	12.05	0.00130	716	268	1.42
CEP250	16.12	0.00045	627	268	1.23
AURKB	11.41	0.00138	141	14	3.35
AURKB	7.68	0.00289	199	14	3.85
CDC20	10.89	0.00152	359	20	4.17
CDC20	8.94	0.00168	688	20	5.11
E2F1	10.54	0.00158	476	43	3.46
E2F1	8.85	0.00174	673	43	3.95
OTX1	10.29	0.00166	31	2	4.27
OTX1	6.25	0.00811	26	2	4.02
SUMO1	9.86	0.00181	2422	2982	−0.3
SUMO1	8.63	0.00188	2624	2982	−0.18
MCM4	9.76	0.00186	1308	336	1.96
MCM4	6.76	0.00535	1327	336	1.98
HGS	7.96	0.00312	2504	1167	1.1
HGS	13.9	0.00066	2441	1167	1.06
KPNA2	7.86	0.00323	1927	689	1.48
KPNA2	10.43	0.00116	2533	689	1.88
UBC	7.85	0.00323	34388	35490	−0.05
UBC	6.63	0.00591	36931	35490	0.06
SPRY2	7.5	0.00376	409	989	−1.27
SPRY2	7.17	0.00399	328	989	−1.59
TOPBP1	7.33	0.00406	799	346	1.21
TOPBP1	6.45	0.00698	875	346	1.34
SIRT7	7.11	0.00456	442	225	0.98
SIRT7	24	0.00012	456	225	1.02
WHSC1	6.67	0.00590	1217	447	1.44
WHSC1	12.06	0.00086	1369	447	1.61
MCM2	6.45	0.00666	1097	174	2.66
MCM2	8.48	0.00200	1351	174	2.96
AURKA	6.4	0.00685	570	86	2.73
AURKA	9.25	0.00154	516	86	2.58
COPS5	6.39	0.00691	1506	1184	0.35
COPS5	10	0.00128	1708	1184	0.53
PCNA	6.29	0.00749	1652	904	0.87
PCNA	15.95	0.00046	2290	904	1.34
BUB1B	6.27	0.00762	179	12	3.89
BUB1B	7.47	0.00327	320	12	4.73
DNMT1	6.02	0.00920	1376	455	1.6
DNMT1	10.63	0.00110	1383	455	1.6

There are two rows with same proteins name while the upper row represents the early stage liver cancer and the lower raw represents the late stage liver cancer.

AvgExp means average expression.

log_2_⁡FC means log_2_⁡ fold change.

**Table 3:** Top 20 proteins of early-stage and late-stage liver cancer.

Protein	CRV	*p* value	Case_AvgExp	Control_AvgExp	log_2_⁡FC
Top 20 proteins of early-stage liver cancer					
APP	46.52	6.09*E* − 06	13724	14041	−0.03
KRTAP4-12	33.47	6.09*E* − 05	1	1	−0.17
ELAVL1	22.99	0.000329	1725	1319	0.39
KRTAP10-1	17.17	0.000756	1	1	0.04
KRTAP10-5	14.73	0.000981	1	1	0.04
H2AFX	14.08	0.001048	608	271	1.17
CDK1	13.69	0.001079	335	34	3.32
PRKDC	12.91	0.001176	4011	1684	1.25
CUL3	12.83	0.001188	1376	1606	−0.22
ESR1	12.81	0.001194	228	1537	−2.76
EZH2	12.22	0.001274	310	49	2.66
CEP250	12.05	0.001298	716	268	1.42
AURKB	11.41	0.001383	141	14	3.35
CDC20	10.89	0.001523	359	20	4.17
E2F1	10.54	0.001584	476	43	3.46
OTX1	10.29	0.001664	31	2	4.27
C19orf66	10.2	0.001688	1551	3166	−1.03
SUMO1	9.86	0.00181	2422	2982	−0.3
MCM4	9.76	0.001859	1308	336	1.96
GRB2	9.19	0.0022	3572	2608	0.45

Top 20 proteins of late stage liver cancer					
ESR1	34.1	4.22*E* − 06	245	1537	−2.65
ELAVL1	29.69	2.53*E* − 05	1791	1319	0.44
UBD	28.54	3.38*E* − 05	20926	1781	3.55
YWHAZ	27.31	4.22*E* − 05	13323	6408	1.06
SIRT7	24	0.000122	456	225	1.02
HDAC5	22.07	0.000152	1696	840	1.01
KRTAP10-5	20.54	0.000224	1	1	0.06
EZH2	19.05	0.00027	394	49	3
ILF2	18.86	0.000283	4111	1737	1.24
CEP250	16.12	0.000452	627	268	1.23
PCNA	15.95	0.00046	2290	904	1.34
SUMO2	15.77	0.00049	3305	2112	0.65
H2AFX	15.43	0.000532	806	271	1.58
HSP90AB1	14.43	0.0006	30949	14629	1.08
HGS	13.9	0.000659	2441	1167	1.06
APP	13.87	0.000659	15083	14041	0.1
WHSC1	12.06	0.000861	1369	447	1.61
SETDB1	11.7	0.000912	1203	497	1.28
TRAF2	11.45	0.000938	665	277	1.26
SMARCA4	11.18	0.000992	2696	1115	1.27
SFN	11.03	0.001026	1008	55	4.19

AvgExp means average expression.

log_2_⁡FC means log_2_⁡ fold change.

**Table 4:** Top 30 proteins of total-stage liver cancer.

Protein	CRV	*p* value	Case_AvgExp	Control_AvgExp	log_2_⁡FC
APP	81.53	<10^−9^	14385	14041	0.03
ELAVL1	3.65*E* + 01	0.000187	1757	1319	0.41
CCDC33	2.82*E* + 01	0.000464	3	1	1.7
UBC	25.19	0.000588	35625	35490	0.01
HIST3H3	24.3	0.000626	1	1	0.02
KRTAP10-1	23.96	0.000643	1	1	0.02
ESR1	23.79	0.000652	236	1537	−2.7
UBD	23.34	0.000682	14701	1781	3.04
H2AFX	17.28	0.001125	705	271	1.38
KRTAP10-5	17.05	0.001133	1	1	0.05
HGS	15.2	0.001329	2473	1167	1.08
TRAF2	14.28	0.001461	581	277	1.07
HSP90AB1	14.16	0.001483	29756	14629	1.02
PCNA	13.58	0.001572	1962	904	1.12
SMARCA4	13.21	0.001611	2546	1115	1.19
TAF6	12.84	0.001636	1075	478	1.17
SUMO2	12.6	0.001704	2968	2112	0.49
UBQLN4	12.59	0.001713	1881	905	1.05
COPS5	12.45	0.001743	1604	1184	0.44
CEP250	12.41	0.001747	673	268	1.33
EZH2	12.15	0.001802	351	49	2.84
CDK1	11.69	0.001892	462	34	3.78
TCF3	11.35	0.00199	980	415	1.24
CDC20	11.28	0.002011	519	20	4.7
MCM2	10.9	0.002126	1220	174	2.81
GRB2	10.64	0.002233	3488	2608	0.42
WHSC1	10.5	0.002288	1291	447	1.53
MYOD1	10.5	0.002292	2	1	1.22
AURKB	10.48	0.002305	169	14	3.61
SUMO1	10.36	0.002373	2520	2982	−0.24

**Table 5:** The intersection of the total-stage liver cancer with our previous result.

Protein	CRV (in this study)	*p* value (in this study)	CRV (previous)	*p* value (previous)
BUB1B	7.58	0.005833	5.5696	0.00064
CDC20	11.28	0.002011	5.1507	0.00109
CDK2	6.9	0.007708	14.069	<1*E* − 5
CUL3	8.16	0.004478	12.9519	<1*E* − 5
E2F1	10.19	0.002497	3.9947	0.00862
ESR1	23.79	0.000652	10.3758	<1*E* − 5
HDAC4	10.11	0.002531	5.8397	0.00048
HGS	15.2	0.001329	4.6929	0.00232
MYC	7.55	0.005884	10.7821	<1*E* − 5
PCNA	13.58	0.001572	15.1438	<1*E* − 5
PRKDC	8.19	0.004418	5.9369	0.00041
SMARCA4	13.21	0.001611	7.7449	0.0001
SUMO1	10.36	0.002373	15.8533	<1*E* − 5
TRAF2	14.28	0.001461	4.7703	0.00207
UBC	25.19	0.000588	137.284	<1*E* − 5

**(a):** 

Rank	Term	Count	Symbol	*p* value
KEGG				
1	hsa04110:Cell cycle	14	E2F1, CDK1, PRKDC, CDC20, MCM2, MCM3, YWHAE, MCM4, CDK2, MCM7, GSK3B, PCNA, BUB1B, MYC	5.96*E* − 13
2	hsa05215:Prostate cancer	7	E2F1, HSP90AB1, GRB2, GSK3B, MAPK3, PTEN, CDK2	2.62*E* − 05
3	hsa04114:Oocyte meiosis	7	CDK1, PPP1CA, MAPK3, CDC20, AURKA, YWHAE, CDK2	8.70*E* − 05
4	hsa03030:DNA replication	5	MCM7, PCNA, MCM2, MCM3, MCM4	9.48*E* − 05
5	hsa05200:Pathways in cancer	10	E2F1, HSP90AB1, TRAF2, GRB2, MSH2, GSK3B, MAPK3, MYC, PTEN, CDK2	2.73*E* − 04
6	hsa05213:Endometrial cancer	5	GRB2, GSK3B, MAPK3, MYC, PTEN	4.03*E* − 04
7	hsa05210:Colorectal cancer	5	GRB2, MSH2, GSK3B, MAPK3, MYC	0.002457
8	hsa05222:Small cell lung cancer	5	E2F1, TRAF2, MYC, PTEN, CDK2	0.002457
9	hsa05214:Glioma	4	E2F1, GRB2, MAPK3, PTEN	0.008946
10	hsa04722:Neurotrophin signaling pathway	5	GRB2, GSK3B, MAPK3, YWHAE, TP73	0.009832
11	hsa04115:p53 signaling pathway	4	CDK1, PTEN, CDK2, TP73	0.011029
12	hsa05220:Chronic myeloid leukemia	4	E2F1, GRB2, MAPK3, MYC	0.014381
13	hsa04914:Progesterone-mediated oocyte maturation	4	HSP90AB1, CDK1, MAPK3, CDK2	0.020705
14	hsa04012:ErbB signaling pathway	4	GRB2, GSK3B, MAPK3, MYC	0.021345
15	hsa04540:Gap junction	4	CDK1, GRB2, MAPK3, TUBA1B	0.022657
16	hsa05219:Bladder cancer	3	E2F1, MAPK3, MYC	0.033366
17	hsa04510:Focal adhesion	5	PPP1CA, GRB2, GSK3B, MAPK3, PTEN	0.047655
18	hsa05223:Non-small cell lung cancer	3	E2F1, GRB2, MAPK3	**0.052694**
19	hsa05221:Acute myeloid leukemia	3	GRB2, MAPK3, MYC	**0.059845**
20	hsa04910:Insulin signaling pathway	4	PPP1CA, GRB2, GSK3B, MAPK3	**0.064636**
21	hsa05218:Melanoma	3	E2F1, MAPK3, PTEN	**0.085159**
22	hsa04662:B cell receptor signaling pathway	3	GRB2, GSK3B, MAPK3	**0.09351**

BioCarte				
1a	h_g1Pathway:Cell Cycle:G_1_/S Check Point	4	E2F1, CDK1, GSK3B, CDK2	0.012616
2a	h_p27Pathway:Regulation of p27 Phosphorylation during Cell Cycle Progression	3	E2F1, NEDD8, CDK2	0.020162
3a	h_p53Pathway:p53 Signaling Pathway	3	E2F1, PCNA, CDK2	0.033691
4a	h_her2Pathway:Role of ERBB2 in Signal Transduction and Oncology	3	GRB2, MAPK3, ESR1	0.049866
5a	h_ptenPathway:PTEN dependent cell cycle arrest and apoptosis	3	GRB2, MAPK3, PTEN	**0.054277**
6a	h_RacCycDPathway:Influence of Ras and Rho proteins on G_1_ to S Transition	3	E2F1, MAPK3, CDK2	**0.063496**
7a	h_cellcyclePathway:Cyclins and Cell Cycle Regulation	3	E2F1, CDK1, CDK2	**0.068295**

The significant pathways via DAVID Bioinformatics database are selected for the 74 significant proteins in carcinogenesis. Bold indicates *p* value > 0.05. Most of them are from the KEGG database, and we add several key pathways from BioCarta.

**(b):** 

GO:term	*p* value	Corrected *p* value	*R*	*T*	*G*	*O*	Term name
(1) Biological processes							
GO:0051320	9.1*E* − 10	1.9*E* − 7	6357	9	12	4	S phase
GO:0000084	9.1*E* − 10	1.9*E* − 7	6357	9	12	4	S phase of mitotic cell cycle
GO:0006267	3.3*E* − 9	7.1*E* − 7	6357	9	16	4	Prereplicative complex assembly
GO:0065004	1.4*E* − 8	3.1*E* − 6	6357	9	68	5	Protein-DNA complex assembly
GO:0000727	2.7*E* − 8	5.8*E* − 6	6357	9	26	4	Double-strand break repair via break-induced replication
GO:0006270	3.7*E* − 8	8.0*E* − 6	6357	9	28	4	DNA-dependent DNA replication initiation
GO:0022616	5.7*E* − 8	1.2*E* − 5	6357	9	31	4	DNA strand elongation
GO:0006271	5.7*E* − 8	1.2*E* − 5	6357	9	31	4	DNA strand elongation involved in DNA replication
GO:0000724	1.3*E* − 7	2.8*E* − 5	6357	9	38	4	Double-strand break repair via homologous recombination
GO:0022402	2.5*E* − 7	5.3*E* − 5	6357	9	445	7	Cell cycle process

(2) Cellular components							
GO:0042555	2.7*E* − 11	1.3*E* − 9	6357	9	6	4	MCM complex
GO:0005656	3.3*E* − 9	1.5*E* − 7	6357	9	16	4	Prereplicative complex
GO:0031261	1.3*E* − 8	6.3*E* − 7	6357	9	22	4	DNA replication preinitiation complex
GO:0031298	2.3*E* − 8	1.0*E* − 6	6357	9	25	4	Replication fork protection complex
GO:0032993	2.9*E* − 7	1.3*E* − 5	6357	9	46	4	Protein-DNA complex
GO:0043234	9.7*E* − 7	4.5*E* − 5	6357	9	1369	9	Protein complex
GO:0044454	4.9*E* − 6	2.3*E* − 4	6357	9	217	5	Nuclear chromosome part
GO:0044451	1.5*E* − 5	7.5*E* − 4	6357	9	275	5	Nucleoplasm part
GO:0044428	1.6*E* − 5	7.7*E* − 4	6357	9	1251	8	Nuclear part
GO:0044427	2.5*E* − 5	0.0011	6357	9	302	5	Chromosomal part

(3) Molecular functions							
GO:0043566	3.8*E* − 10	2.6*E* − 8	6357	9	85	6	Structure-specific DNA binding
GO:0043138	5.6*E* − 9	3.7*E* − 7	6357	9	18	4	3′-5′ DNA helicase activity
GO:0004003	2.7*E* − 8	1.8*E* − 6	6357	9	26	4	ATP-dependent DNA helicase activity
GO:0003682	3.3*E* − 8	2.2*E* − 6	6357	9	80	5	Chromatin binding
GO:0003688	4.9*E* − 8	3.3*E* − 6	6357	9	30	4	DNA replication origin binding
GO:0003678	1.4*E* − 7	9.9*E* − 6	6357	9	39	4	DNA helicase activity
GO:0003697	2.4*E* − 7	1.6*E* − 5	6357	9	44	4	Single-stranded DNA binding
GO:0016887	4.9*E* − 7	3.3*E* − 5	6357	9	278	6	ATPase activity
GO:0043140	5.5*E* − 7	3.7*E* − 5	6357	9	13	3	ATP-dependent 3′-5′ DNA helicase activity
GO:0008094	1.3*E* − 6	9.1*E* − 5	6357	9	67	4	DNA-dependent ATPase activity

*R*: number of genes in reference set.

*T*: number of genes in test set.

*G*: number of genes annotated by given term in reference set.

*O*: number of genes annotated by given term in test set.

**(a):** 

Rank	Term	Count	Symbol	*p* value
KEGG				
1	hsa04110:Cell cycle	11	E2F1, CCNB1, CDK1, CDKN2A, PCNA, BUB1B, PRKDC, CDC20, MCM2, MCM4, MYC	4.90*E* − 11
2	hsa03030:DNA replication	4	POLD1, PCNA, MCM2, MCM4	5.24*E* − 04
3	hsa05220:Chronic myeloid leukemia	4	E2F1, CDKN2A, GRB2, MYC	0.004415
4	hsa04114:Oocyte meiosis	4	CCNB1, CDK1, CDC20, AURKA	0.01273
5	hsa05219:Bladder cancer	3	E2F1, CDKN2A, MYC	0.015099
6	hsa05223:Non-small cell lung cancer	3	E2F1, CDKN2A, GRB2	0.024285
7	hsa05214:Glioma	3	E2F1, CDKN2A, GRB2	0.03234
8	hsa04115:p53 signaling pathway	3	CCNB1, CDK1, CDKN2A	0.037212
9	hsa03430:Mismatch repair	2	POLD1, PCNA	0.09922

BioCarta				
1a	h_cellcyclePathway:Cyclins and Cell Cycle Regulation	4	E2F1, CCNB1, CDK1, CDKN2A	0.002246
2a	h_srcRPTPPathway:Activation of Src by Protein-tyrosine phosphatase alpha	3	CCNB1, CDK1, GRB2	0.004
3a	h_g2Pathway:Cell Cycle:G_2_/M Checkpoint	3	CCNB1, CDK1, PRKDC	0.025668
4a	h_g1Pathway:Cell Cycle:G_1_/S Check Point	3	E2F1, CDK1, CDKN2A	0.039621
5a	h_ptc1Pathway:Sonic Hedgehog (SHH) Receptor Ptc1 Regulates cell cycle	2	CCNB1, CDK1	**0.075537**

The significant pathways via DAVID Bioinformatics database are selected for the 43 significant proteins in carcinogenesis. Bold indicates *p* value > 0.05. Most of them are from the KEGG database, and we add several key pathways from BioCarta.

**(b):** 

GO:term	*p* value	Corrected *p* value	*R*	*T*	*G*	*O*	Term name
(1) Biological processes							
GO:0051320	1.9*E* − 5	0.0028	6357	4	12	2	S phase
GO:0000084	1.9*E* − 5	0.0028	6357	4	12	2	S phase of mitotic cell cycle
GO:0006267	3.5*E* − 5	0.0052	6357	4	16	2	Prereplicative complex assembly
GO:0000727	9.6*E* − 5	0.0142	6357	4	26	2	Double-strand break repair via break-induced replication
GO:0006270	1.1*E* − 4	0.0165	6357	4	28	2	DNA-dependent DNA replication initiation
GO:0022616	1.3*E* − 4	0.0203	6357	4	31	2	DNA strand elongation
GO:0006271	1.3*E* − 4	0.0203	6357	4	31	2	DNA strand elongation involved in DNA replication
GO:0000724	2.0*E* − 4	0.0306	6357	4	38	2	Double-strand break repair via homologous recombination
GO:0000725	3.1*E* − 4	0.0470	6357	4	47	2	Recombinational repair
GO:0022403	3.4*E* − 4	0.0515	6357	4	286	3	Cell cycle phase

(2) Cellular components							
GO:0042555	4.4*E* − 6	1.3*E* − 4	6357	4	6	2	MCM complex
GO:0005656	3.5*E* − 5	0.0010	6357	4	16	2	Prereplicative complex
GO:0031261	6.8*E* − 5	0.0020	6357	4	22	2	DNA replication preinitiation complex
GO:0031298	8.8*E* − 5	0.0026	6357	4	25	2	Replication fork protection complex
GO:0032993	3.0*E* − 4	0.0091	6357	4	46	2	Protein-DNA complex
GO:0000151	5.7*E* − 4	0.0171	6357	4	63	2	Ubiquitin ligase complex
GO:0043234	0.0021	0.0643	6357	4	1369	4	Protein complex
GO:0033597	0.0025	0.0754	6357	4	4	1	Mitotic checkpoint complex
GO:0031463	0.0031	0.0942	6357	4	5	1	Cul3-RING ubiquitin ligase complex
GO:0043596	0.0062	0.1883	6357	4	10	1	Nuclear replication fork

(3) Molecular functions							
GO:0043138	4.5*E* − 5	0.0021	6357	4	18	2	3′-5′ DNA helicase activity
GO:0004003	9.6*E* − 5	0.0046	6357	4	26	2	ATP-dependent DNA helicase activity
GO:0003688	1.2*E* − 4	0.0061	6357	4	30	2	DNA replication origin binding
GO:0003678	2.1*E* − 4	0.0104	6357	4	39	2	DNA helicase activity
GO:0003697	2.7*E* − 4	0.0133	6357	4	44	2	Single-stranded DNA binding
GO:0008094	6.4*E* − 4	0.0310	6357	4	67	2	DNA-dependent ATPase activity
GO:0003682	9.2*E* − 4	0.0443	6357	4	80	2	Chromatin binding
GO:0043566	0.0010	0.0500	6357	4	85	2	Structure-specific DNA binding
GO:0004842	0.0010	0.0500	6357	4	85	2	Ubiquitin-protein ligase activity
GO:0070035	0.0010	0.0511	6357	4	86	2	Purine NTP-dependent helicase activity

*R*: number of genes in reference set.

*T*: number of genes in test set.

*G*: number of genes annotated by given term in reference set.

*O*: number of genes annotated by given term in test set.

**(a):** 

Rank	Term	Count	Symbol	*p* value
KEGG				
1	hsa04110:Cell cycle	16	E2F1, CDK1, YWHAZ, YWHAB, CDC20, SFN, MCM2, MCM3, CDK4, MCM4, CDK2, MCM7, PCNA, YWHAQ, BUB1B, CCNA2	3.90*E* − 14
2	hsa04114:Oocyte meiosis	8	CDK1, YWHAZ, YWHAB, YWHAQ, CDC20, AURKA, PPP1CC, CDK2	3.62*E* − 05
3	hsa03030:DNA replication	5	MCM7, PCNA, MCM2, MCM3, MCM4	2.35*E* − 04
4	hsa04115:p53 signaling pathway	4	CDK1, SFN, CDK4, CDK2	0.020496
5	hsa03040:Spliceosome	5	SNRPB, THOC4, LSM2, SF3A2, SF3B4	0.022722
6	hsa05222:Small cell lung cancer	4	E2F1, TRAF2, CDK4, CDK2	0.035432
7	hsa04914:Progesterone-mediated oocyte maturation	4	HSP90AB1, CDK1, CCNA2, CDK2	0.037607
8	hsa00310:Lysine degradation	3	SETDB1, WHSC1, EHMT2	**0.055046**
9	hsa05200:Pathways in cancer	7	E2F1, HSP90AB1, TRAF2, MSH2, BIRC5, CDK4, CDK2	**0.061178**

BioCarte				
1a	h_p53Pathway:p53 Signaling Pathway	4	E2F1, PCNA, CDK4, CDK2	0.003378
2a	h_cellcyclePathway:Cyclins and Cell Cycle Regulation	4	E2F1, CDK1, CDK4, CDK2	0.010334
3a	h_g1Pathway:Cell Cycle:G_1_/S Check Point	4	E2F1, CDK1, CDK4, CDK2	0.015615
4a	h_rbPathway:RB Tumor Suppressor/Checkpoint Signaling in response to DNA damage	3	CDK1, CDK4, CDK2	0.019987
5a	h_ranMSpathway:Role of Ran in mitotic spindle regulation	3	RAN, AURKA, KPNA2	0.019987
6a	h_g2Pathway:Cell Cycle:G_2_/M Checkpoint	3	CDK1, YWHAQ, BRCA1	**0.067509**
7a	h_RacCycDPathway:Influence of Ras and Rho proteins on G_1_ to S Transition	3	E2F1, CDK4, CDK2	**0.072809**

The significant pathways via DAVID Bioinformatics database are selected for the 74 significant proteins in carcinogenesis. Bold indicates *p* value > 0.05. Most of them are from the KEGG database, and we add several key pathways from BioCarta.

**(b):** 

GO:term	*p* value	Corrected *p* value	*R*	*T*	*G*	*O*	Term name
(1) Biological processes							
GO:0051320	1.5*E* − 9	3.6*E* − 7	6357	10	12	4	S phase
GO:0000084	1.5*E* − 9	3.6*E* − 7	6357	10	12	4	S phase of mitotic cell cycle
GO:0006267	5.5*E* − 9	1.3*E* − 6	6357	10	16	4	Prereplicative complex assembly
GO:0000727	4.5*E* − 8	1.1*E* − 5	6357	10	26	4	Double-strand break repair via break-induced replication
GO:0006270	6.2*E* − 8	1.5*E* − 5	6357	10	28	4	DNA-dependent DNA replication initiation
GO:0022616	9.5*E* − 8	2.3*E* − 5	6357	10	31	4	DNA strand elongation
GO:0006271	9.5*E* − 8	2.3*E* − 5	6357	10	31	4	DNA strand elongation involved in DNA replication
GO:0000724	2.2*E* − 7	5.4*E* − 5	6357	10	38	4	Double-strand break repair via homologous recombination
GO:0006260	5.1*E* − 7	1.2*E* − 4	6357	10	120	5	DNA replication
GO:0000725	5.3*E* − 7	1.2*E* − 4	6357	10	47	4	Recombinational repair

(2) Cellular components							
GO:0042555	4.6*E* − 11	2.2*E* − 9	6357	10	6	4	MCM complex
GO:0005656	5.5*E* − 9	2.6*E* − 7	6357	10	16	4	Prereplicative complex
GO:0031261	2.2*E* − 8	1.0*E* − 6	6357	10	22	4	DNA replication preinitiation complex
GO:0031298	3.8*E* − 8	1.8*E* − 6	6357	10	25	4	Replication fork protection complex
GO:0032993	4.8*E* − 7	2.3*E* − 5	6357	10	46	4	Protein-DNA complex
GO:0044454	2.3*E* − 4	0.0113	6357	10	217	4	Nuclear chromosome part
GO:0044451	5.8*E* − 4	0.0280	6357	10	275	4	Nucleoplasm part
GO:0044428	7.7*E* − 4	0.0370	6357	10	1251	7	Nuclear part
GO:0044427	8.3*E* − 4	0.0400	6357	10	302	4	Chromosomal part
GO:0043234	0.0013	0.0656	6357	10	1369	7	Protein complex

(3) Molecular functions							
GO:0043138	9.3*E* − 9	6.4*E* − 7	6357	10	18	4	3′-5′ DNA helicase activity
GO:0004003	4.5*E* − 8	3.1*E* − 6	6357	10	26	4	ATP-dependent DNA helicase activity
GO:0003688	8.3*E* − 8	5.7*E* − 6	6357	10	30	4	DNA replication origin binding
GO:0043566	9.0*E* − 8	6.2*E* − 6	6357	10	85	5	Structure-specific DNA binding
GO:0003678	2.4*E* − 7	1.7*E* − 5	6357	10	39	4	DNA helicase activity
GO:0003697	4.0*E* − 7	2.8*E* − 5	6357	10	44	4	Single-stranded DNA binding
GO:0043140	7.9*E* − 7	5.4*E* − 5	6357	10	13	3	ATP-dependent 3′-5′ DNA helicase activity
GO:0008094	2.2*E* − 6	1.5*E* − 4	6357	10	67	4	DNA-dependent ATPase activity
GO:0003682	4.6*E* − 6	3.1*E* − 4	6357	10	80	4	Chromatin binding
GO:0070035	6.1*E* − 6	4.2*E* − 4	6357	10	86	4	Purine NTP-dependent helicase activity

*R*: number of genes in reference set.

*T*: number of genes in test set.

*G*: number of genes annotated by given term in reference set.

*O*: number of genes annotated by given term in test set.

## References

[1] Seyfried T. N., Shelton L. M. (2010). Cancer as a metabolic disease. *Nutrition & Metabolism*.

[2] Hanahan D., Weinberg R. A. (2000). The hallmarks of cancer. *Cell*.

[3] Hanahan D., Weinberg R. A. (2011). Hallmarks of cancer: the next generation. *Cell*.

[4] Preziosi L. (2003). *Cancer Modelling and Simulation*.

[5] Barillot E. (2013). *Computational Systems Biology of Cancer*.

[6] Cesario A., Marcus F. B. (2011). *Cancer Systems Biology, Bioinformatics and Medicine: Research and Clinical Applications*.

[7] Wang E. (2010). *Cancer Systems Biology*.

[8] Yan Q. (2010). *Systems Biology in Drug Discovery and Development: Methods and Protocols*.

[9] Juan H.-F., Huang H.-C. (2012). *Systems Biology: Applications in Cancer-Related Research*.

[10] Li C.-W., Chu Y., Chen B. (2006). Construction and clarification of dynamic gene regulatory network of cancer cell cycle via microarray data. *Cancer Informatics*.

[11] Chu L.-H., Chen B.-S. (2008). Comparisons of robustness and sensitivity between cancer and normal cells by microarray data. *Cancer Informatics*.

[12] Chu L.-H., Chen B.-S. (2008). Construction of a cancer-perturbed protein-protein interaction network for discovery of apoptosis drug targets. *BMC Systems Biology*.

[13] Wang Y.-C., Chen B.-S. (2011). A network-based biomarker approach for molecular investigation and diagnosis of lung cancer. *BMC Medical Genomics*.

[14] Tu C.-T., Chen B.-S. (2013). New measurement methods of network robustness and response ability via microarray data. *PLoS ONE*.

[15] Wong Y. H., Chen R.-H., Chen B.-S. (2014). Core and specific network markers of carcinogenesis from multiple cancer samples. *Journal of Theoretical Biology*.

[16] Wong Y., Li C., Chen B. (2014). Evolution of network biomarkers from early to late stage bladder cancer samples. *BioMed Research International*.

[17] Jemal A., Bray F., Center M. M., Ferlay J., Ward E., Forman D. (2011). Global cancer statistics. *CA Cancer Journal for Clinicians*.

[18] Altekruse S. F., McGlynn K. A., Reichman M. E. (2009). Hepatocellular carcinoma incidence, mortality, and survival trends in the United States from 1975 to 2005. *Journal of Clinical Oncology*.

[19] Li Y., Tang Z.-Y., Hou J.-X. (2012). Hepatocellular carcinoma: insight from animal models. *Nature Reviews Gastroenterology and Hepatology*.

[20] Marquardt J. U., Galle P. R., Teufel A. (2012). Molecular diagnosis and therapy of hepatocellular carcinoma (HCC): an emerging field for advanced technologies. *Journal of Hepatology*.

[21] Aravalli R. N., Cressman E. N. K., Steer C. J. (2013). Cellular and molecular mechanisms of hepatocellular carcinoma: an update. *Archives of Toxicology*.

[22] Sun B. C., Karin M. (2012). Obesity, inflammation, and liver cancer. *Journal of Hepatology*.

[23] Villanueva A., Hernandez-Gea V., Llovet J. M. (2013). Medical therapies for hepatocellular carcinoma: a critical view of the evidence. *Nature Reviews Gastroenterology and Hepatology*.

[24] Zhang Y., Wang S., Li D. (2011). A systems Biology-Based classifier for hepatocellular carcinoma diagnosis. *PLoS ONE*.

[25] Zhang Y. Q., Guo X., Wang D. (2014). A systems biology-based investigation into the therapeutic effects of Gansui Banxia Tang on reversing the imbalanced network of hepatocellular carcinoma. *Scientific Reports*.

[26] D'Alessandro L. A., Meyer R., Klingmüller U. (2013). Hepatocellular carcinoma: a systems biology perspective. *Frontiers in Physiology*.

[27] Chen L., Liu R., Liu Z.-P., Li M., Aihara K. (2012). Detecting early-warning signals for sudden deterioration of complex diseases by dynamical network biomarkers. *Scientific Reports*.

[28] Liu R., Wang X., Aihara K., Chen L. (2014). Early diagnosis of complex diseases by molecular biomarkers, network biomarkers, and dynamical network biomarkers. *Medicinal Research Reviews*.

[29] Liu R., Yu X., Liu X., Xu D., Aihara K., Chen L. (2014). Identifying critical transitions of complex diseases based on a single sample. *Bioinformatics*.

[30] Wang Y.-C., Chen B.-S. (2011). A network-based biomarker approach for molecular investigation and diagnosis of lung cancer. *BMC Medical Genomics*.

[31] Liu K.-Q., Liu Z.-P., Hao J.-K., Chen L., Zhao X.-M. (2012). Identifying dysregulated pathways in cancers from pathway interaction networks. *BMC Bioinformatics*.

[32] Chatr-Aryamontri A., Breitkreutz B.-J., Heinicke S. (2013). The BioGRID interaction database: 2013 update. *Nucleic Acids Research*.

[33] Bland J. M., Altman D. G. (1995). Multiple significance tests: the Bonferroni method. *British Medical Journal*.

[34] Johansson R. (1993). *System Modeling and Identification*.

[35] Pagano M., Gauvreau K. (2000). *Principles of Biostatistics*.

[36] Kanehisa M. (2013). Molecular network analysis of diseases and drugs in KEGG. *Methods in Molecular Biology*.

[37] Satoh J.-I. (2012). Molecular network of microRNA targets in Alzheimer's disease brains. *Experimental Neurology*.

[38] Huang D. W., Sherman B. T., Lempicki R. A. (2009). Systematic and integrative analysis of large gene lists using DAVID bioinformatics resources. *Nature Protocols*.

[39] Huang D. W., Sherman B. T., Lempicki R. A. (2009). Bioinformatics enrichment tools: paths toward the comprehensive functional analysis of large gene lists. *Nucleic Acids Research*.

[40] Wang J., Huang Q., Liu Z.-P. (2011). NOA: a novel Network Ontology Analysis method. *Nucleic Acids Research*.

[41] Zhang C., Wang J., Hanspers K., Xu D., Chen L., Pico A. R. (2013). NOA: a cytoscape plugin for network ontology analysis. *Bioinformatics*.

[42] Williams G. H., Stoeber K. (2012). The cell cycle and cancer. *Journal of Pathology*.

[43] Lu J., He M.-L., Wang L. (2011). miR-26a inhibits cell growth and tumorigenesis of nasopharyngeal carcinoma through repression of EZH2. *Cancer Research*.

[44] Cheung K.-F., Zhao J., Hao Y. (2013). CITED2 is a novel direct effector of peroxisome proliferator-activated receptor *γ* in suppressing hepatocellular carcinoma cell growth. *Cancer*.

[45] Dituri F., Mazzocca A., Lupo L. (2012). PI3K class IB controls the cell cycle checkpoint promoting cell proliferation in hepatocellular carcinoma. *International Journal of Cancer*.

[46] Furuta M., Kozaki K.-I., Tanimoto K. (2013). The tumor-suppressive miR-497-195 cluster targets multiple cell-cycle regulators in hepatocellular carcinoma. *PLoS ONE*.

[47] Zhang L., Guo Y., Li B. (2013). Identification of biomarkers for hepatocellular carcinoma using network-based bioinformatics methods. *European Journal of Medical Research*.

[48] Terret M.-E., Chaigne A., Verlhac M.-H. (2013). Mouse oocyte, a paradigm of cancer cell. *Cell Cycle*.

[49] Macheret M., Halazonetis T. D. (2015). DNA replication stress as a hallmark of cancer. *Annual Review of Pathology: Mechanisms of Disease*.

[50] Ma L., Teruya-Feldstein J., Weinberg R. A. (2007). Tumour invasion and metastasis initiated by microRNA-10b in breast cancer. *Nature*.

[51] Gao P., Xing A.-Y., Zhou G.-Y. (2013). The molecular mechanism of microRNA-145 to suppress invasion-metastasis cascade in gastric cancer. *Oncogene*.

[52] Negrini S., Gorgoulis V. G., Halazonetis T. D. (2010). Genomic instability—an evolving hallmark of cancer. *Nature Reviews Molecular Cell Biology*.

[53] Hornberg J. J., Bruggeman F. J., Westerhoff H. V., Lankelma J. (2006). Cancer: a systems biology disease. *BioSystems*.

[54] Soussi T., Legros Y., Lubin R., Ory K., Schlichtholz B. (1994). Multifactorial analysis of p53 alteration in human cancer: a review. *International Journal of Cancer*.

[55] Ueda H., Ullrich S. J., Gangemi J. D. (1995). Functional inactivation but not structural mutation of p53 causes liver cancer. *Nature Genetics*.

[56] Fujimoto A., Totoki Y., Abe T. (2012). Whole-genome sequencing of liver cancers identifies etiological influences on mutation patterns and recurrent mutations in chromatin regulators. *Nature Genetics*.

[57] Jurica M. S., Moore M. J. (2003). Pre-mRNA splicing: awash in a sea of proteins. *Molecular Cell*.

[58] Zhou Z., Licklider L. J., Gygi S. P., Reed R. (2002). Comprehensive proteomic analysis of the human spliceosome. *Nature*.

[59] Pillai R. S., Grimmler M., Meister G. (2003). Unique Sm core structure of U7 snRNPs: assembly by a specialized SMN complex and the role of a new component, Lsm11, in histone RNA processing. *Genes and Development*.

[60] Agranat-Tamir L., Shomron N., Sperling J., Sperling R. (2014). Interplay between pre-mRNA splicing and microRNA biogenesis within the supraspliceosome. *Nucleic Acids Research*.

[61] Kaida D., Motoyoshi H., Tashiro E. (2007). Spliceostatin A targets SF3b and inhibits both splicing and nuclear retention of pre-mRNA. *Nature Chemical Biology*.

[62] Kotake Y., Sagane K., Owa T. (2007). Splicing factor SF3b as a target of the antitumor natural product pladienolide. *Nature Chemical Biology*.

[63] van Alphen R. J., Wiemer E. A. C., Burger H., Eskens F. A. L. M. (2009). The spliceosome as target for anticancer treatment. *British Journal of Cancer*.

[64] Tian M., Cheng H., Wang Z. (2015). Phosphoproteomic analysis of the highly-metastatic hepatocellular carcinoma cell line, MHCC97-H. *International Journal of Molecular Sciences*.

[65] Huang Y., Yang X., Zhao F. (2014). Overexpression of Dickkopf-1 predicts poor prognosis for patients with hepatocellular carcinoma after orthotopic liver transplantation by promoting cancer metastasis and recurrence. *Medical Oncology*.

[66] Whitley A. L. P., San Jose C., Hernandez N. (2009). *A Comparison of Next Generation Sequencing and Microarrays for Transcriptome Expression Profiling*.

