# Supplementary material for: Applying NGS Data to Find Evolutionary Network Biomarkers from the Early and Late Stages of Hepatocellular Carcinoma
Source: Biomed Res Int. 2015 Aug 20;2015:391475. doi: 10.1155/2015/391475 (PMC4558430; doi:10.1155/2015/391475)
Supplement: Supplementary file 1 — Supplementary material S.1 uses the Maximum Likelihood Method to do the parameter identification of regression model in equation (1). S.2 uses the AIC and student's t-test to calculate the p-values of association abilities, and detect the system model order and determine the significance of the model parameters. Table S3: (a) The 43 identified significant proteins of early stage liver cancer. (b) The 80 identified significant proteins of late stage liver cancer. (c) The 74 identified significant proteins of total stage liver cancer. [file 391475.f1.zip › 391475.f1/mat.391475.v1.docx]

**Supplementary Materials**

## S.1 Parameter Identification of Regression Model in Equation (1) by Maximum Likelihood Method

Equation (1) can be written as the following requiring form

(S1) where denotes the regression vector which can be obtained from microarray data, is the parameter vector to be estimated. Suppose that there are *m* samples, then it is easy to acquired values of for . In this case, equation (S1) for different samples can be represented as the following vector form.

(S2) where ,

For simplicity, it can be represented as follows.

(S3) where .

In equation (S3), the noise for different samples was regarded as independent random variables of normal distribution with zero mean and unknown variance , i.e., , and , where *I* is the identity matrix. The probability density function of is given as follows.

( S4)

From equation (S4), we can obtain the likelihood function

(S5)

Maximum likelihood estimation method aims at finding and to maximize the likelihood function in equation (S5). In order to simplify the computation, it is practical to take the logarithm of the likelihood function, which yields the following log-likelihood function:

(S6) where and are the *n*-th element of and in (S3), respectively.

By the maximum likelihood parameter estimation method, we expect the log-likelihood function to have the maximum at and . The necessary conditions for the maximum likelihood estimates and must conform to the following two equations.

(S7)

The estimated parameters and are shown below,

(S8)

(S9)

where and can be obtained from the microarray in the rough PPIN. Since there are two data sets of microarray data, two association parameters for cancer and non-cancer were separately identified.

## S.2 Determination of significant protein associations by AIC and Student’s t-test

When association parameters of all the proteins in rough PPIN were identified as equation (2), significant protein associations were determined by parameter estimates of their association abilities. In order to determine whether the association was significant or not, Akaike Information Criterion (AIC) and Student’s t-test, which is used to calculate the *p*-values of the association abilities, are employed to detect the system model order (or the number of model parameters) and determine the significance of our model parameters. The AIC, a method for model order detection, attempts to include both the estimated residual variance and model complexity in one statistic. AIC decreases as residual variance decreases, and increases as the number of parameters increases. As the expected residual variance decreases with increasing parameter numbers for excessive model complexity, a minimum should appear near the correct parameter number. Thus, the AIC criterion, in which estimated parameters were obtained above, was used to select model structure. Due to computation efficiency, it is impractical to compute the AIC statistics for all possible regression models. Here, the stepwise regression method which combines forward selection method and backward elimination method was applied to compute the AIC statistics. Once the estimated association parameters were examined using the AIC model detection criteria, the student’s t-test was employed to calculate the *p*-values for the association abilities under the null hypothesis to determine the significant protein associations. The *p*-values computed were then adjusted by Bonferroni correction to avoid a lot of spurious positives. The associations which adjusted *p*-value ≤ 0.05 were determined as significant associations and were preserved in the protein association network.

Briefly, we use the AIC method to obtain how many system orders, which mean the numbers of interactions, in the dynamic system of the association abilities (model). We use the above maximum likelihood estimate method to identify the parameter and then employ AIC and student t-test to calculate *p-*values of association abilities for determining the significant PPIs for the target protein *i* by pruning the insignificant PPIs.
