# Supplementary material for: Applying NGS Data to Find Evolutionary Network Biomarkers from the Early and Late Stages of Hepatocellular Carcinoma
Source: Biomed Res Int. 2015 Aug 20;2015:391475. doi: 10.1155/2015/391475 (PMC4558430; doi:10.1155/2015/391475)
Supplement: Supplementary file 1 — Supplementary material S.1 uses the Maximum Likelihood Method to do the parameter identification of regression model in equation (1). S.2 uses the AIC and student's t-test to calculate the p-values of association abilities, and detect the system model order and determine the significance of the model parameters. Table S3: (a) The 43 identified significant proteins of early stage liver cancer. (b) The 80 identified significant proteins of late stage liver cancer. (c) The 74 identified significant proteins of total stage liver cancer. [file 391475.f1.zip › 391475.f1/Table S3.docx]

Table S3: (a) The 43 identified significant proteins of early stage liver cancer.

| **Network marker of early stage liver cancer** | | | | | |
| --- | --- | --- | --- | --- | --- |
| **Protein** | **CRV** | ***p*-value** | **Cancer_AvgExp** | **Control_AvgExp** | **log_2_FC** |
| APP | 46.52 | 6.09E-06 | 13724 | 14041 | -0.03 |
| KRTAP4-12 | 33.47 | 6.09E-05 | 1 | 1 | -0.17 |
| ELAVL1 | 22.99 | 0.000329 | 1725 | 1319 | 0.39 |
| KRTAP10-1 | 17.17 | 0.000756 | 1 | 1 | 0.04 |
| KRTAP10-5 | 14.73 | 0.000981 | 1 | 1 | 0.04 |
| H2AFX | 14.08 | 0.001048 | 608 | 271 | 1.17 |
| CDK1 | 13.69 | 0.001079 | 335 | 34 | 3.32 |
| PRKDC | 12.91 | 0.001176 | 4011 | 1684 | 1.25 |
| CUL3 | 12.83 | 0.001188 | 1376 | 1606 | -0.22 |
| ESR1 | 12.81 | 0.001194 | 228 | 1537 | -2.76 |
| EZH2 | 12.22 | 0.001274 | 310 | 49 | 2.66 |
| CEP250 | 12.05 | 0.001298 | 716 | 268 | 1.42 |
| AURKB | 11.41 | 0.001383 | 141 | 14 | 3.35 |
| CDC20 | 10.89 | 0.001523 | 359 | 20 | 4.17 |
| E2F1 | 10.54 | 0.001584 | 476 | 43 | 3.46 |
| OTX1 | 10.29 | 0.001664 | 31 | 2 | 4.27 |
| C19orf66 | 10.2 | 0.001688 | 1551 | 3166 | -1.03 |
| SUMO1 | 9.86 | 0.00181 | 2422 | 2982 | -0.3 |
| MCM4 | 9.76 | 0.001859 | 1308 | 336 | 1.96 |
| GRB2 | 9.19 | 0.0022 | 3572 | 2608 | 0.45 |
| GNE | 8.86 | 0.002352 | 2403 | 6093 | -1.34 |
| TBC1D16 | 8.49 | 0.002669 | 483 | 145 | 1.74 |
| HGS | 7.96 | 0.00312 | 2504 | 1167 | 1.1 |
| KPNA2 | 7.86 | 0.00323 | 1927 | 689 | 1.48 |
| UBC | 7.85 | 0.00323 | 34388 | 35490 | -0.05 |
| SPRY2 | 7.5 | 0.00376 | 409 | 989 | -1.27 |
| TOPBP1 | 7.33 | 0.004059 | 799 | 346 | 1.21 |
| SIRT7 | 7.11 | 0.004564 | 442 | 225 | 0.98 |
| PLSCR4 | 6.96 | 0.00496 | 649 | 1881 | -1.53 |
| UBR5 | 6.93 | 0.005021 | 1953 | 954 | 1.03 |
| HMGA1 | 6.92 | 0.005046 | 1779 | 539 | 1.72 |
| POLD1 | 6.78 | 0.005454 | 637 | 228 | 1.48 |
| WHSC1 | 6.67 | 0.005905 | 1217 | 447 | 1.44 |
| MYC | 6.6 | 0.006155 | 1318 | 3114 | -1.24 |
| FANCD2 | 6.51 | 0.006466 | 196 | 30 | 2.7 |
| MCM2 | 6.45 | 0.006661 | 1097 | 174 | 2.66 |
| AURKA | 6.4 | 0.006849 | 570 | 86 | 2.73 |
| COPS5 | 6.39 | 0.00691 | 1506 | 1184 | 0.35 |
| PCNA | 6.29 | 0.007489 | 1652 | 904 | 0.87 |
| BUB1B | 6.27 | 0.007617 | 179 | 12 | 3.89 |
| CCNB1 | 6.25 | 0.00777 | 517 | 56 | 3.22 |
| DNMT1 | 6.02 | 0.009202 | 1376 | 455 | 1.6 |
| CDKN2A | 5.98 | 0.009421 | 350 | 24 | 3.85 |

AvgExp means average expression

Log_2_FC means log_2_ fold change

**(b) The 80 identified significant proteins of late stage liver cancer.**

| **Network marker of early stage liver cancer** | | | | | |
| --- | --- | --- | --- | --- | --- |
| **Protein** | **CRV** | ***p*-value** | **Cancer_AvgExp** | **Control_AvgExp** | **log_2_FC** |
| ESR1 | 34.1 | 4.22E-06 | 245 | 1537 | -2.65 |
| ELAVL1 | 29.69 | 2.53E-05 | 1791 | 1319 | 0.44 |
| UBD | 28.54 | 3.38E-05 | 20926 | 1781 | 3.55 |
| YWHAZ | 27.31 | 4.22E-05 | 13323 | 6408 | 1.06 |
| SIRT7 | 24 | 0.000122 | 456 | 225 | 1.02 |
| HDAC5 | 22.07 | 0.000152 | 1696 | 840 | 1.01 |
| KRTAP10-5 | 20.54 | 0.000224 | 1 | 1 | 0.06 |
| EZH2 | 19.05 | 0.00027 | 394 | 49 | 3 |
| ILF2 | 18.86 | 0.000283 | 4111 | 1737 | 1.24 |
| CEP250 | 16.12 | 0.000452 | 627 | 268 | 1.23 |
| PCNA | 15.95 | 0.00046 | 2290 | 904 | 1.34 |
| SUMO2 | 15.77 | 0.00049 | 3305 | 2112 | 0.65 |
| H2AFX | 15.43 | 0.000532 | 806 | 271 | 1.58 |
| HSP90AB1 | 14.43 | 0.0006 | 30949 | 14629 | 1.08 |
| HGS | 13.9 | 0.000659 | 2441 | 1167 | 1.06 |
| APP | 13.87 | 0.000659 | 15083 | 14041 | 0.1 |
| WHSC1 | 12.06 | 0.000861 | 1369 | 447 | 1.61 |
| SETDB1 | 11.7 | 0.000912 | 1203 | 497 | 1.28 |
| TRAF2 | 11.45 | 0.000938 | 665 | 277 | 1.26 |
| SMARCA4 | 11.18 | 0.000992 | 2696 | 1115 | 1.27 |
| SFN | 11.03 | 0.001026 | 1008 | 55 | 4.19 |
| SF3B4 | 10.86 | 0.00106 | 2513 | 953 | 1.4 |
| UBQLN4 | 10.84 | 0.001068 | 2041 | 905 | 1.17 |
| DNMT1 | 10.63 | 0.001102 | 1383 | 455 | 1.6 |
| KPNA2 | 10.43 | 0.001161 | 2533 | 689 | 1.88 |
| SHC1 | 10.4 | 0.001174 | 5970 | 2660 | 1.17 |
| CDK1 | 10.31 | 0.001191 | 596 | 34 | 4.15 |
| MSH2 | 10.29 | 0.001199 | 630 | 195 | 1.7 |
| CCDC33 | 10.06 | 0.001267 | 6 | 1 | 2.49 |
| COPS5 | 10 | 0.001284 | 1708 | 1184 | 0.53 |
| AURKA | 9.25 | 0.001541 | 516 | 86 | 2.58 |
| PKM2 | 9.24 | 0.001541 | 8072 | 1272 | 2.67 |
| ECT2 | 9.11 | 0.001609 | 743 | 60 | 3.64 |
| SF3A2 | 9.02 | 0.00163 | 1328 | 642 | 1.05 |
| MCM7 | 8.96 | 0.001664 | 2627 | 773 | 1.77 |
| CDC20 | 8.94 | 0.001681 | 688 | 20 | 5.11 |
| BLM | 8.9 | 0.001702 | 129 | 13 | 3.37 |
| PCK1 | 8.88 | 0.001731 | 14695 | 90420 | -2.62 |
| E2F1 | 8.85 | 0.00174 | 673 | 43 | 3.95 |
| TCF3 | 8.75 | 0.001795 | 1019 | 415 | 1.3 |
| ILF3 | 8.64 | 0.001871 | 4132 | 2027 | 1.03 |
| SUMO1 | 8.63 | 0.001879 | 2624 | 2982 | -0.18 |
| SNRPB | 8.55 | 0.001964 | 3212 | 1505 | 1.09 |
| COPS6 | 8.52 | 0.001976 | 2331 | 1649 | 0.5 |
| CDK2 | 8.49 | 0.001997 | 748 | 455 | 0.72 |
| MCM3 | 8.48 | 0.001997 | 2319 | 679 | 1.77 |
| MCM2 | 8.48 | 0.001997 | 1351 | 174 | 2.96 |
| TK1 | 8.4 | 0.002031 | 1215 | 182 | 2.74 |
| EHMT2 | 8.23 | 0.002179 | 1535 | 516 | 1.57 |
| ACTL6A | 7.73 | 0.002813 | 788 | 383 | 1.04 |
| AURKB | 7.68 | 0.002889 | 199 | 14 | 3.85 |
| MAGEA11 | 7.65 | 0.00296 | 7 | 1 | 2.86 |
| BUB1B | 7.47 | 0.003273 | 320 | 12 | 4.73 |
| YWHAB | 7.27 | 0.003695 | 5498 | 3779 | 0.54 |
| YWHAQ | 7.26 | 0.003712 | 5255 | 3378 | 0.64 |
| CCT3 | 7.21 | 0.003877 | 10442 | 4188 | 1.32 |
| CRMP1 | 7.19 | 0.003902 | 100 | 25 | 1.99 |
| SPRY2 | 7.17 | 0.003991 | 328 | 989 | -1.59 |
| CCT6A | 7.05 | 0.004345 | 4746 | 2158 | 1.14 |
| CCNA2 | 7 | 0.004438 | 738 | 35 | 4.4 |
| TUBG1 | 6.93 | 0.004726 | 1246 | 515 | 1.27 |
| ACLY | 6.91 | 0.004772 | 3449 | 1329 | 1.38 |
| RAN | 6.81 | 0.005106 | 4213 | 2067 | 1.03 |
| MCM4 | 6.76 | 0.005351 | 1327 | 336 | 1.98 |
| BRCA1 | 6.71 | 0.005612 | 335 | 148 | 1.18 |
| CDK4 | 6.64 | 0.005874 | 2390 | 1148 | 1.06 |
| NUP107 | 6.64 | 0.005874 | 748 | 366 | 1.03 |
| HCFC1 | 6.64 | 0.005874 | 2087 | 947 | 1.14 |
| UBC | 6.63 | 0.005908 | 36931 | 35490 | 0.06 |
| PPP1CC | 6.59 | 0.006144 | 2713 | 1553 | 0.8 |
| MYOD1 | 6.54 | 0.00641 | 4 | 1 | 1.89 |
| HIST3H3 | 6.48 | 0.006769 | 1 | 1 | 0.05 |
| TOPBP1 | 6.45 | 0.006985 | 875 | 346 | 1.34 |
| OTUB2 | 6.4 | 0.007247 | 134 | 33 | 2.03 |
| LSM2 | 6.39 | 0.00731 | 780 | 368 | 1.08 |
| FBF1 | 6.28 | 0.007935 | 99 | 25 | 2.02 |
| OTX1 | 6.25 | 0.008112 | 26 | 2 | 4.02 |
| UBE2S | 6.21 | 0.008323 | 419 | 115 | 1.87 |
| BIRC5 | 6.13 | 0.00897 | 374 | 10 | 5.2 |
| THOC4 | 6.12 | 0.009041 | 1128 | 527 | 1.1 |

**(c) The 74 identified significant proteins of total stage liver cancer.**

| **Network marker of early stage liver cancer** | | | | | |
| --- | --- | --- | --- | --- | --- |
| **Protein** | **CRV** | ***p*-value** | **Cancer_AvgExp** | **Control_AvgExp** | **log_2_FC** |
| APP | 81.53 | <10^-9 | 14385 | 14041 | 0.03 |
| ELAVL1 | 3.65E+01 | 0.000187 | 1757 | 1319 | 0.41 |
| CCDC33 | 2.82E+01 | 0.000464 | 3 | 1 | 1.7 |
| UBC | 25.19 | 0.000588 | 35625 | 35490 | 0.01 |
| HIST3H3 | 24.3 | 0.000626 | 1 | 1 | 0.02 |
| KRTAP10-1 | 23.96 | 0.000643 | 1 | 1 | 0.02 |
| ESR1 | 23.79 | 0.000652 | 236 | 1537 | -2.7 |
| UBD | 23.34 | 0.000682 | 14701 | 1781 | 3.04 |
| H2AFX | 17.28 | 0.001125 | 705 | 271 | 1.38 |
| KRTAP10-5 | 17.05 | 0.001133 | 1 | 1 | 0.05 |
| HGS | 15.2 | 0.001329 | 2473 | 1167 | 1.08 |
| TRAF2 | 14.28 | 0.001461 | 581 | 277 | 1.07 |
| HSP90AB1 | 14.16 | 0.001483 | 29756 | 14629 | 1.02 |
| PCNA | 13.58 | 0.001572 | 1962 | 904 | 1.12 |
| SMARCA4 | 13.21 | 0.001611 | 2546 | 1115 | 1.19 |
| TAF6 | 12.84 | 0.001636 | 1075 | 478 | 1.17 |
| SUMO2 | 12.6 | 0.001704 | 2968 | 2112 | 0.49 |
| UBQLN4 | 12.59 | 0.001713 | 1881 | 905 | 1.05 |
| COPS5 | 12.45 | 0.001743 | 1604 | 1184 | 0.44 |
| CEP250 | 12.41 | 0.001747 | 673 | 268 | 1.33 |
| EZH2 | 12.15 | 0.001802 | 351 | 49 | 2.84 |
| CDK1 | 11.69 | 0.001892 | 462 | 34 | 3.78 |
| TCF3 | 11.35 | 0.00199 | 980 | 415 | 1.24 |
| CDC20 | 11.28 | 0.002011 | 519 | 20 | 4.7 |
| MCM2 | 10.9 | 0.002126 | 1220 | 174 | 2.81 |
| GRB2 | 10.64 | 0.002233 | 3488 | 2608 | 0.42 |
| WHSC1 | 10.5 | 0.002288 | 1291 | 447 | 1.53 |
| MYOD1 | 10.5 | 0.002292 | 2 | 1 | 1.22 |
| AURKB | 10.48 | 0.002305 | 169 | 14 | 3.61 |
| SUMO1 | 10.36 | 0.002373 | 2520 | 2982 | -0.24 |
| E2F1 | 10.19 | 0.002497 | 572 | 43 | 3.72 |
| HDAC4 | 10.11 | 0.002531 | 272 | 114 | 1.26 |
| ZNF581 | 9.62 | 0.002838 | 322 | 142 | 1.19 |
| DNMT1 | 9.5 | 0.00294 | 1380 | 455 | 1.6 |
| ILF2 | 9.32 | 0.003072 | 3684 | 1737 | 1.08 |
| MCM4 | 9.01 | 0.003315 | 1317 | 336 | 1.97 |
| TP73 | 8.99 | 0.003332 | 63 | 5 | 3.67 |
| TK1 | 8.26 | 0.004308 | 868 | 182 | 2.25 |
| SCNM1 | 8.26 | 0.004312 | 533 | 236 | 1.18 |
| PRKDC | 8.19 | 0.004418 | 3974 | 1684 | 1.24 |
| CUL3 | 8.16 | 0.004478 | 1493 | 1606 | -0.11 |
| PTEN | 8.16 | 0.004482 | 1960 | 2595 | -0.4 |
| SETDB1 | 8.16 | 0.004482 | 1108 | 497 | 1.16 |
| SIRT7 | 8.06 | 0.004644 | 449 | 225 | 1 |
| HCFC1 | 8.02 | 0.004734 | 2169 | 947 | 1.2 |
| KIF2C | 7.94 | 0.004951 | 341 | 12 | 4.86 |
| KPNA2 | 7.87 | 0.005126 | 2222 | 689 | 1.69 |
| MAPK3 | 7.86 | 0.005138 | 910 | 516 | 0.82 |
| YWHAE | 7.85 | 0.005164 | 7003 | 8837 | -0.34 |
| UHRF1 | 7.8 | 0.005296 | 237 | 17 | 3.81 |
| SMC2 | 7.66 | 0.005628 | 569 | 187 | 1.61 |
| OTX1 | 7.64 | 0.005663 | 29 | 2 | 4.15 |
| MCM7 | 7.61 | 0.005803 | 2291 | 773 | 1.57 |
| BUB1B | 7.58 | 0.005833 | 247 | 12 | 4.36 |
| MSH2 | 7.56 | 0.005876 | 616 | 195 | 1.66 |
| MYC | 7.55 | 0.005884 | 1804 | 3114 | -0.79 |
| COPS6 | 7.51 | 0.006016 | 2257 | 1649 | 0.45 |
| ECT2 | 7.43 | 0.006187 | 582 | 60 | 3.29 |
| LGR4 | 7.35 | 0.006459 | 3322 | 3581 | -0.11 |
| AURKA | 7.26 | 0.006711 | 543 | 86 | 2.66 |
| ZGPAT | 7.1 | 0.007111 | 1592 | 4828 | -1.6 |
| KDM1A | 7.1 | 0.00712 | 937 | 751 | 0.32 |
| MCM3 | 7.07 | 0.007231 | 2166 | 679 | 1.67 |
| NEDD8 | 6.97 | 0.007542 | 2113 | 1647 | 0.36 |
| CENPA | 6.95 | 0.007576 | 104 | 5 | 4.4 |
| CDK2 | 6.9 | 0.007708 | 706 | 455 | 0.63 |
| TUBA1B | 6.9 | 0.007708 | 11580 | 5032 | 1.2 |
| GSK3B | 6.87 | 0.007818 | 1215 | 874 | 0.48 |
| SF3B4 | 6.83 | 0.007959 | 2261 | 953 | 1.25 |
| TUBG1 | 6.78 | 0.008159 | 1171 | 515 | 1.19 |
| AVPI1 | 6.77 | 0.00821 | 498 | 1243 | -1.32 |
| PPP1CA | 6.7 | 0.0085 | 3631 | 3023 | 0.26 |
| MDFI | 6.59 | 0.008994 | 77 | 12 | 2.71 |
| TULP3 | 6.5 | 0.009446 | 478 | 217 | 1.14 |
